# Changes of Neolithic subsistence in south Hangzhou Bay coast, eastern China: An adaptive strategy to landscape processes

**DOI:** 10.3389/fpls.2022.1000583

**Published:** 2022-09-20

**Authors:** Jinqi Dai, Lanjie Deng, Dan Feng, Xiaoshuang Zhao, Huimin Wang, Xueming Li, Li Xiao, Xiaoyu Zhang, Jing Chen, Maotian Li, Zhongyuan Chen, Yan Liu, Qianli Sun

**Affiliations:** ^1^State Key Laboratory of Estuarine and Coastal Research, East China Normal University, Shanghai, China; ^2^School of Geography and Ocean Science, Nanjing University, Nanjing, China

**Keywords:** pollen, phytoliths, Holocene, geomorphic evolution, rice exploitation

## Abstract

The transition from hunting and gathering to agricultural subsistence is a striking feature of the Neolithic revolution worldwide. Known as the cradle of a series of representative Neolithic cultures, south Hangzhou Bay (SHB) witnessed substantial changes in both landscape and human subsistence during the Holocene, yet the relationship between them was not well established. Here, we combined archaeobotanical results from sediment cores with archaeological findings to illustrate the subsistence changes during the Neolithic regime in the context of the landscape process in SHB. Our result showed that SHB was inundated by marine transgression 8,200 years ago without significant human imprints. At 8,200–7,600 cal yr. BP, the initial coastal wetland formation at locations with the semi-enclosed landscape would have facilitated the activities of hunting-gathering, incipient rice cultivation, and collecting seafood if accessible. Pollen and phytoliths evidence from multiple sediment cores in the Yaojiang Valley (YJV) suggested a desalinization process of wetland in the following hundreds of years. This amelioration of the environment had favored the intermittent rice cultivation at various locations in the YJV, where archaeological evidence was absent. Since 7,000–6,600 cal yr. BP, as freshwater wetland expanded with coastal progradation, a wide variety of food resources became available. Meanwhile, rice domestication began to serve as a crucial food supplement as evidenced by both microfossil results and archaeological findings. With the expansion of the coastal plain after 5,500 cal yr. BP, rice farming became widespread and rice consumption was increasingly important in the diet, as supported by discoveries of upgraded farming tools, abundant rice remains, and ancient rice paddies. Above all, the change of subsistence from hunting-gathering to rice farming exhibited an adaptive strategy in response to landscape evolution from an initial marine-influenced setting to a later coastal plain.

## Introduction

During the post-glacial global warming period, agricultural economies featuring crop cultivation and animal domestication began to appear (Bellwood and Diamond, 2005; Bar-Yosef, 2011; Fuller et al., 2014). The transformation from reliance on hunting-gathering to farming economies is one of the most phenomenal changes in the history of human civilization (Childe, 1936; Diamond, 2002). In particular, the cultivation, management, and domestication of rice by Neolithic ancients are critical in the transition of the ecosystem from entirely natural to a situation severely affected by human communities. Meanwhile, changes in the spectrum of food resources and their internal mechanism are essential issues in geo-archaeology over the past decades (Bellwood, 2005; Pan and Yuan, 2018, 2019; Hu, 2021). However, the impetus for humans to shift from hunting-gathering to domestication remains uncertain (Childe, 1936; Bar-Yosef, 2011; Fuller et al., 2014). On various spatio-temporal scales, environmental change has been considered a very indispensable element for the rise and fall of prehistoric cultures and the subsistence strategies that have interacted and co-evolved with cultural development (Childe, 1936; Bar-Yosef and Belfer-Cohen, 1992; Lucas et al., 2017; Zheng et al., 2017; Liu et al., 2021).

In the past few years, the south Hangzhou Bay (SHB) coast of eastern China has been recognized as a core region for the initiation, development, and dispersal of rice cultivation and domestication through a series of Neolithic cultures on the coastal lowlands. The prolonged Neolithic history traced back to 11,000 years ago has provided solid records of the human-landscape interactions. The relationship between Holocene sea-level changes and subsequent depositional evolution in the SHB has been outlined based on numerous sediment cores (Lin et al., 2005; Zhang et al., 2014; Liu et al., 2021). Previous studies have established the marine transgressive and retrogressive sequence by microfossil and geochemical evidence (Liu et al., 2014; Dai et al., 2018). However, most of these studies paid attention to the universality and general changes of the environmental change on a broader spatial scale, yet differences in local topographic and geomorphologic evolution were not well-presented in detail. In particular, how specific locations responded to the regional environmental change was not clearly illustrated, especially for those locations that were selected by the early settlers. The differences in the evolution of local environments may have a non-negligible impact on the strategy of human activities.

Recent research on plant remains and microfossils obtained from archaeological sites have provided new clues on how Neolithic humans would react in case of environmental changes. These studies have made in-depth explorations into the development of Neolithic cultures and the strategy of food selection. Nevertheless, the succession of sedimentary records and plant traces obtained from archaeological sites might have been compromised by human interferences, constraining a comprehensive understanding of the human-landscape relation.

In this paper, we synthesized palaeo-environmental records from our new sediment cores (TJA and YJ1504) and published records close to archaeological sites to complement the archaeological findings. Aside from a series of AMS-^14^C datings, we also applied grain size, phytolith, pollen, foraminifera, and algae analysis to ensure a detailed reconstruction of environmental evolution and human activities. In addition, by incorporating published records that cover various spatial and temporal information on the human-landscape interactions in the SHB, we hope to present new insights into the differentiated environmental changes on the local and regional level, as well as the associated subsistence patterns and adaptive strategies chosen by the Neolithic people. This study would shed light on the evolution of Neolithic subsistence changes corresponding to various environmental processes and would provide a new understanding of the vivid human-landscape combat of our ancestors.

## Materials and methods

### Physical setting

**Figure 1:**
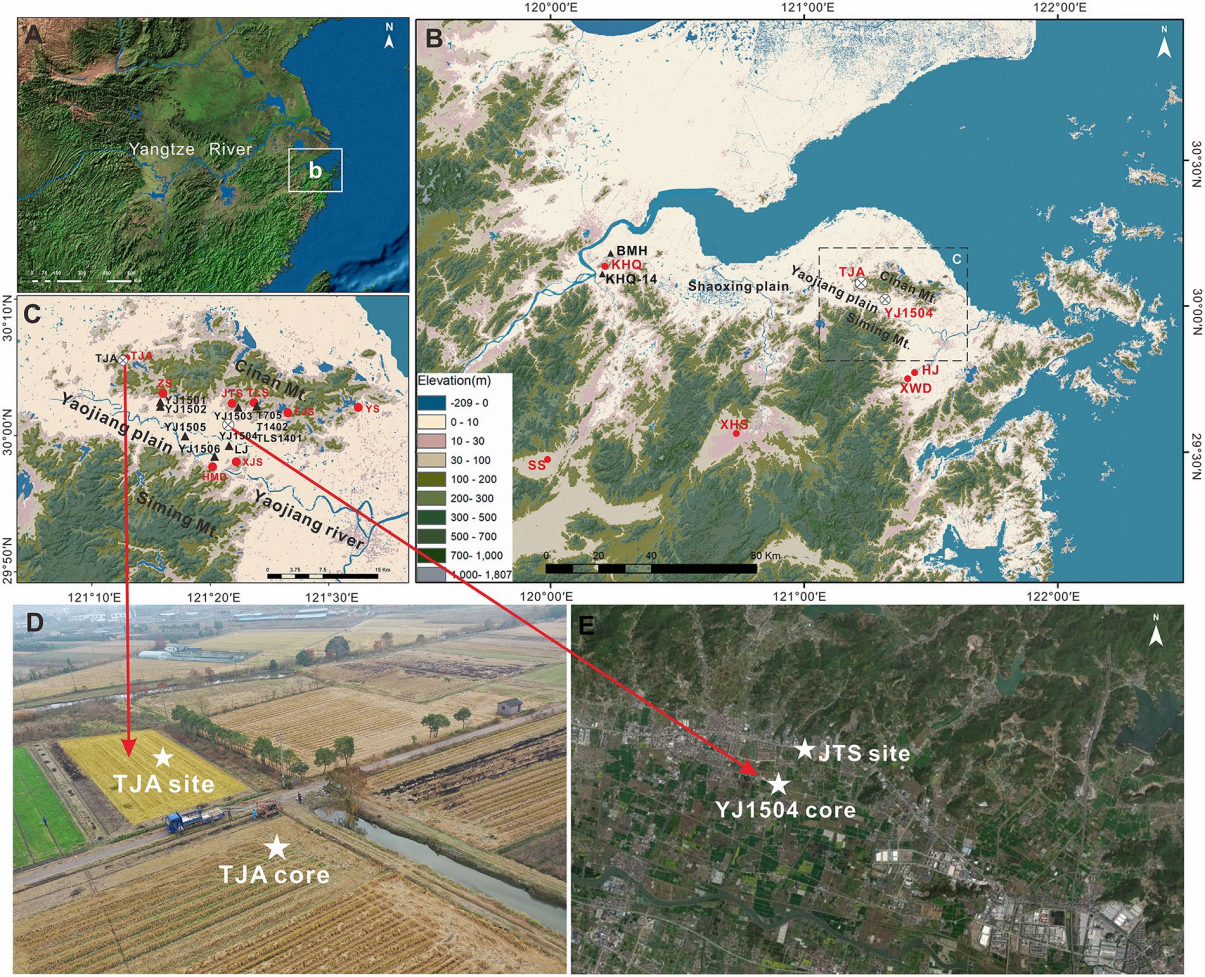
**(A)** Geographic background and location of the study area. **(B)** Location of the lower Yangtze river. **(C)** Location of the Yaojiang valley. **(D)** Location of the TJA core. **(E)** Location of the YJ1504 core. Red dots represent archaeological sites and black triangles represent cores mentioned in the present study. The location of the core sites of TJA and YJ1504 is marked with the black and white dots. SS, Shangshan site; XHS, Xiaohuangshan site; KHQ, Kuahuqiao site; JTS, Jingtoushan site; TJA, Tongjia’ao site; TLS, Tianluoshan site; HMD, Hemudu site; XJS, Xiangjiashan site; FJS, Fujiashan site; YS, Yushan site; HJ, Hejia site; XWD, Xiawangdu site; BMH, Baimahu core; LJ, Luojiang core.

The Yaojiang Valley is located at the eastern part of the Ningshao Plain on the eastern coast of China, with an average elevation of about 3 m (Figure 1). Low mountains and hills are distributed on the north and south sides of the valley, with Siming Mountain in the south and Cinan Mountain in the north. The Yaojiang River passes through the Yaojiang Valley from east to west to join the Fenghua River on the Ningbo Plain and, form the Yongjiang River which flows into the Hangzhou Bay. This area is often affected by typhoons and storm surges due to its location in the path of the Pacific typhoon and the low-lying coastal topography.

The study area sits in the subtropical region of southeastern China. The average temperature in January is ~4 °C, the average temperature in July is ~28 °C, and the average annual precipitation is about 1,100 mm (Ningbo Chorography Codification Committee, 1995). The study area is rich in vegetation, mainly in the subtropical evergreen broad-leaved forest. The main group species and dominant species include Fagaceae, Lauraceae, Magnoliaceae, and Theaceae. Wetlands and lakes are generally distributed in the plain area, mainly covered with freshwater herbs including *Typha*, *Phragmites,* and Chenopodiaceae; aquatic plants mainly include *Nelumbo*, *Zizania latifolia*, *Sagittaria*, *Azolla*, *Trapa,* and *Euryale ferox*. The mountainous area is composed of evergreen and deciduous trees, including *Quercus*, *Castanopsis*, *Morus*, *Juglans*, *Liquidambar*, as well as *Pinus massoniana* and *Cyclocarya*, etc. The higher altitudes of the study area are covered with coniferous forest species including pines (Wu, 1980).

### Core retrieval

Two sediment cores were newly drilled to reconstruct the palaeo-environmental and vegetational changes and human activities. We used a real-time kinematic (RTK, model: ZGP800A) measuring system to obtain the ground elevation of the cores referring to the National Huanghai Datum. Core TJA (1.65 m above mean sea level) was obtained at the Tongjia’ao archaeological site from the north part of the YJV in 2019 using an Acker drill rig (Figure 1). Sedimentological, chronological, and microfossil analyses including phytolith, pollen, charcoal, dinoflagellate, and foraminifera were employed to provide new pieces of evidence of sedimentary environments and food strategies in the study area during the Neolithic period.

Core YJ1504 was 1 km south to Jingtoushan archaeological site in the northwest part of the YJV, with a ground elevation of 1.12 m above mean sea level (Figure 1). The lithology of core YJ1504 was published in Liu et al. (2018), and we analyzed phytoliths and algae in the present study to decode the history the local environmental change and human activity. In addition, information on sediment cores and archaeological findings in the SHB were extracted from published literature and incorporated into the present study to help establish regional palaeo-environmental and archaeological contexts.

### Site descriptions

#### Tongjia’ao archaeological site

The Tongjia’ao site was located on a small alluvial plain semi-enclosed by hills only several hundred meters nearby. Archaeological excavations on the Tongjia’ao site indicated human occupation at the site since *ca.* 7,000 cal yr. BP, which equals to the first phase of Hemudu Culture (Ningbo Municipal Institute of Cultural Relics and Archaeology, Cixi Museum, 2012). Bone farming tools, *Si* (plough), were unearthed from the early cultural layers (Supplementary Table S1) in addition to multiple potteries related to food and water storage. More importantly, a paved road was discovered at the site, though its function was unclear yet.

#### Jingtoushan archaeological site

The Jingtoushan site was located in the YJV, and its cultural layers were buried 7–11 m below the ground surface. Human occupation at the site was dated back to 8,300–7,800 cal yr. BP, which was much earlier than the Hemudu culture, and is contemporary to the occupation at the Kuahuqiao site. Remains of freshwater plants, fruits and animal bones surfaced at the site, as well as marine sourced fish bones, bivalve shells and oyster shells (Supplementary Table S1). Rice remains and macrofossil evidence also indicated the usage of rice at the site (Zhejiang Provincial Institute of Cultural Relics and Archaeology, Ningbo Institute of Cultural Heritage Management, Yuyao Hemudu Site Museum, 2021).

### Ages and calibrations

In total, 10 samples of different materials, including plant fragments (PF), peat and organic matter (OM), were taken from core TJA for accelerator mass spectrometry (AMS) radiocarbon dating. In YJ1504, three dates were collected from Liu et al. (2018) and five additional dates were obtained using PF, OM, charcoal (CH), and shell. The AMS ^14^C dating was performed at the Institute of Earth Environment, Chinese Academy of Sciences (IEECAS) in Xi’an, China and the Beta Analytic radiocarbon laboratory for dating. All these ^14^C dates were calibrated by Calib Rev. 7.0.4 using the IntCal 13 and Marine 13 curve to standardize the chronology from different cores (Reimer et al., 2013).

### Grain-size analysis

In total, 320 and 86 samples of the core TJA and core YJ1504 were collected for grain size analyses. The sampling intervals were 2 cm of the core TJA and 10 cm of the core YJ1504. Samples were first dried before being treated with HCl (10%) and H_2_O_2_ (10%) to remove carbonates and humic acid, respectively. Finally, Na(PO_3_)_6_ was added to disperse the sediment sample before testing. Grain-size frequency distributions were made with a Beckman Coulter Laser Diffraction Particle Size Analyzer (LS13320), which has a measurement range of 0.02–2,000 μm. All these pretreatment and measurement were performed at the State Key Laboratory of Estuarine and Coastal Research (SKLEC) in Shanghai, China.

### Microfossil analysis

A series of microfossil analyses were employed to reconstruct the palaeo-environmental change at the studied sites, with emphasis on the impact of marine influence over time.

### Phytoliths analysis in TJA

In total, 52 samples were collected from core TJA at approx. 10 cm intervals at 10–490 cm and 20 cm intervals at 500–580 cm. A wet digestion method was used to extract phytoliths: about 5–6 g of dry sediment was placed in a tube; 10 ml of H_2_O_2_ was added to get rid of the OM and 5 ml of HCl was added to remove the carbonates; phytoliths were then extracted by ZnBr_2_ (2.35 g/cm^3^); finally, the recovered phytoliths were preserved in with neutral balsam. A minimum of 300 phytoliths were counted for each sample with a Nikon microscope at 400× magnification. All the phytoliths were classified according to modern references and published criteria (Wang and Lu, 1992; International Committee for Phytolith T, 2019). In addition, three rice phytoliths types, including rice bulliforms, paralleled bilobates, and double-peaked glume cells were distinguished (Gu et al., 2013).

### Foraminifera analysis in TJA

There were 65 samples taken from the TJA core for foraminifera identification. The sampling interval is 10 cm at depths of 10–130 cm and 220–630 cm and 20 cm at depths of 140–220 cm and 630–740 cm. A total of 25 g of dry soil was used for each sample for the pre-treatment with reference to Wang et al. (1985). The identification of foraminifera was performed using a Nikon microscope under 400× magnification and the abundances were given in grains/g.

### Pollen, dinoflagellate, and charcoal analysis in TJA

A total of 48 samples were taken for pollen, dinoflagellate and charcoal analyses with an interval of 10 cm at depths of 20–450 cm, and 20 cm at depths of 460–560 cm. Five gram of soil for each sample was dried for 2 days. The pre-treatment followed the method of Moore et al. (1991) and the identification of pollen was conducted following Wang et al. (1995) and Tang et al. (2019a). Pollen, spores, dinoflagellate, and charcoal were identified and counted with a Leica DM3000 at 400× magnification. At least 250 pollen grains were counted from each sample. The pollen, dinoflagellate, and charcoal concentration was calculated by adding *a Lycopodium* tablet to each sample.

According to previous investigations, Poaceae pollen was divided into three groups (>40 μm, 35–39 μm, and < 35 μm). Poaceae pollen with a diameter > 35 μm (>40 μm and 35–39 μm) was related to rice cultivation, and more likely, Poaceae pollen >40 μm hinted the well-managed rice cultivation. Poaceae pollen of <35 μm indicated the interferences of more non-rice weedy grasses (Yang et al., 2012; Liu et al., 2016). In addition, *Quercus* pollen was divided into two types: the evergreen type and the deciduous type based on the empirical rule (pollen size and decorations) from previous research (Wang and Pu, 2004; Liu et al., 2007). Charcoal counting was done along with pollen identification, following the method of (Millspaugh and Whitlock, 1995). Macro-charcoal (>100 μm) was exclusively identified to indicate the local fire (Li et al., 2010).

### Phytoliths analysis in YJ1504

A total of 42 samples were taken from the YJ1504 core for phytolith identification. Forty-one samples were taken from core YJ1504 at about 20 cm intervals at 75–875 cm. Only one sample was taken from 10 to 25 cm and no samples were collected from depths of 25–75 cm because this layer was contaminated by artificial fill. A wet digestion method was applied to the extraction of phytoliths of YJ1504 as well.

### Algae analysis in YJ1504

To outline the general history of marine influence, eight samples were taken at 50–750 cm in YJ1504. About 3 g of each sample were taken into a beaker after drying and smashing; 10% NaOH, 20% HCl and 40% HF were added separately to eliminate humic acid, carbonate, and silicate; the sediment suspensions were filtered by a 10 μm mesh in an ultrasonic bath and mounted on glass microscopic slides; and the pretreated samples were prepared and identified by an optical microscope under 400× magnification. The algae were mainly divided into freshwater, brackish, or saltwater groups. The former is represented by *Concentricystes and Pediastrum*, and the latter is represented by Dinoflagellates (Mao et al., 2011).

### Neolithic context of the SHB

The SHB coast was concentrated with many Neolithic settlements since the middle Holocene, and abundant human imprints were well preserved in the archaeological sites and sedimentary archives. The information on the ages of Neolithic sites, food resources, and tools from previous publications and excavation reports was reorganized in the present study, and to help generate a comprehensive vision of the evolutionary history of human subsistence (Supplementary Table S1).

## Results

### Lithology and stratigraphy

**Figure 2:**
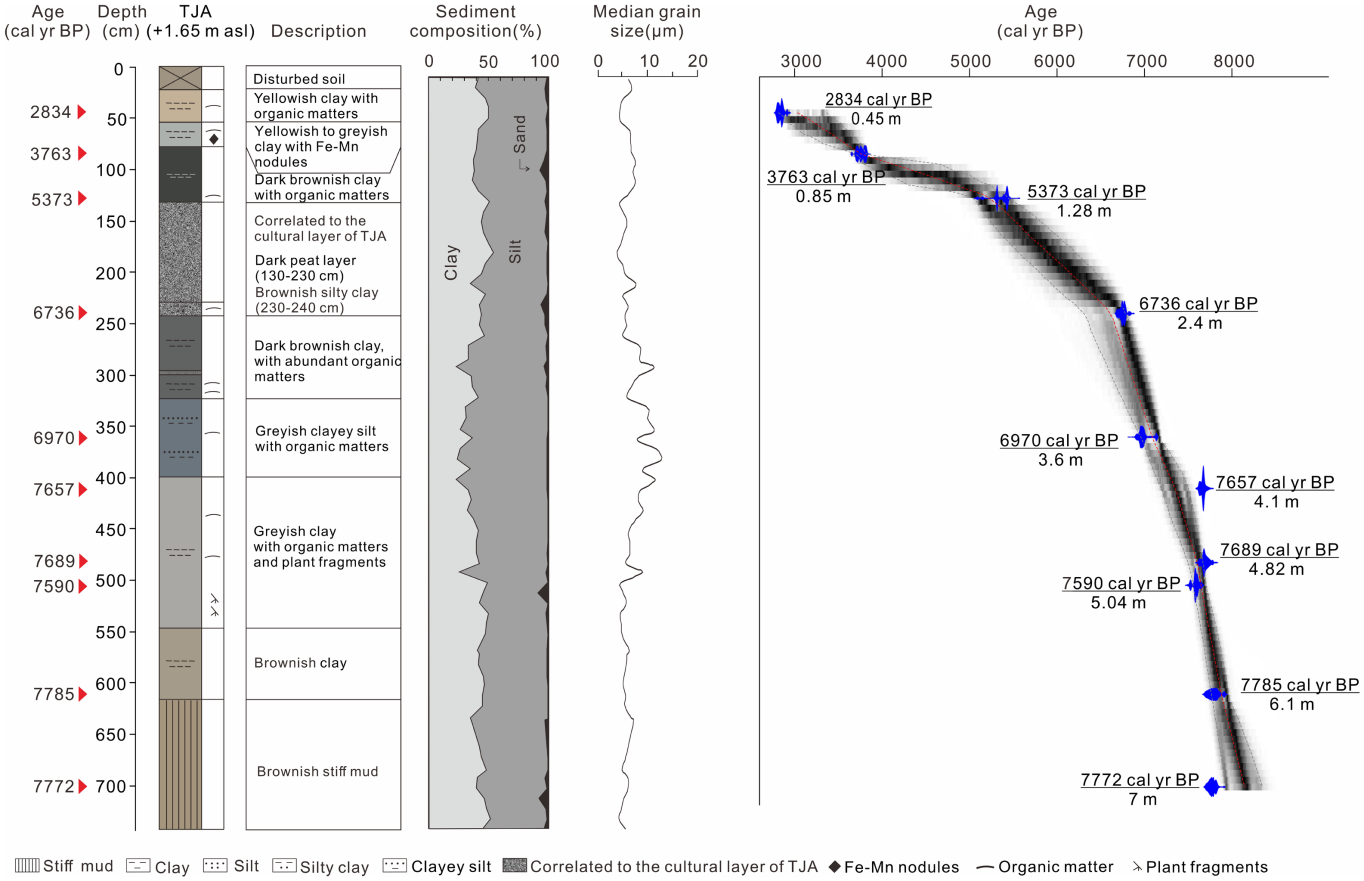
Sediment lithology, grain-size distributions, and age model of core TJA.

**Figure 3:**
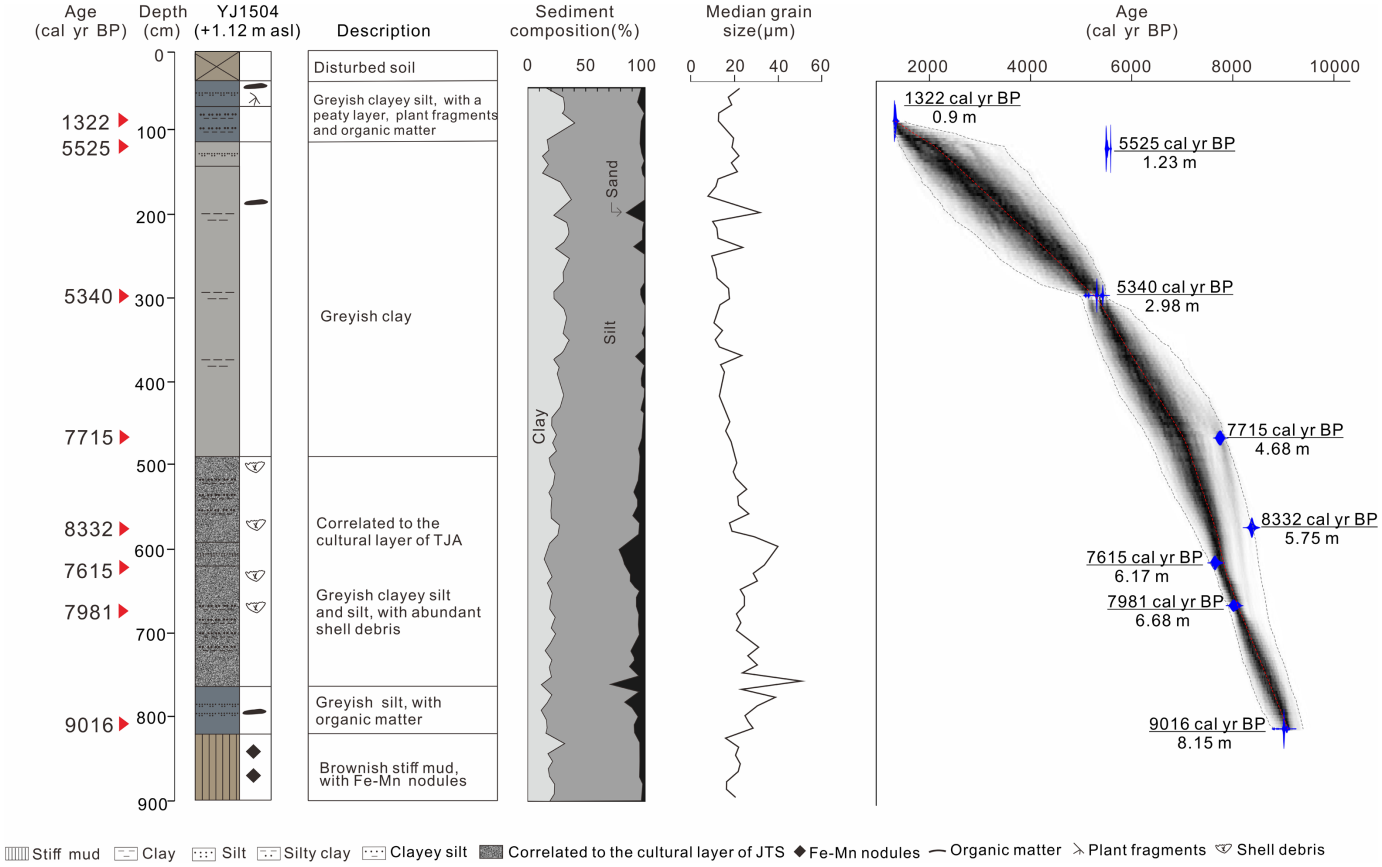
Sediment lithology, grain-size distributions and age model of core YJ1504.

All 18 ^14^C determinations and lithology of TJA and YJ1504 are shown in Table 1; Figures 2, 3.

**Table 1:** Detailed information on AMS^14^C datings in cores TJA and YJ1504.

Core	Depth/cm	Lab code	Material	^14^C age (yr BP)	Error	δ^13^C (‰)	Calibration age 2δ (cal yr. BP)	Medium	Weighted mean
TJA	45	XA50368	OM	2,755	17	−26.7	2,786–2,881	2,842	2,834
	85	XA50370	Peat	3,480	18	−29.6	3,696–3,829	3,761	3,763
	128	Beta-563273	Peat	4,610	30	−27.9	5,288–5,458	5,404	5,373
	240	XA50372	PF	5,932	20	−28.0	6,678–6,794	6,755	6,736
	360	XA51838	PF	6,105	20	−26.3	6,900–7,148	6,973	6,970
	410	XA51839	PF	6,840	20	−22.5	7,617–7,697	7,671	7,657
	482	Beta-571489	PF	6,860	30	−28.3	7,618–7,759	7,687	7,689
	504	Beta-571490	PF	6,720	30	−27.9	7,514–7,656	7,588	7,590
	610	Beta-567008	PF	6,960	40	−25.2	7,690–7,923	7,790	7,785
	700	XA50375	PF	6,950	20	−27.6	7,706–7,837	7,775	7,772
YJ1504	90	XA15945	OM	1,420	20	−28.45	1,295–1,348	1,319	1,322
	123	XA51834	PF	4,805	15	−27.7	5,483–5,591	5,505	5,525
	298	XA51833	PF	4,595	25	−44.5	5,286–5,446	5,316	5,340
	468	XA51855	Shell	7,290	25	−5.4	7,548–7,877	7,702	7,715
	575	XA51856	Shell	7,920	30	−5.1	8,154–8,509	8,325	8,332
	617	XA15926	Shell	7,190	40	−8.28	7,445–7,784	7,609	7,615
	668	XA15927	Shell	7,575	40	−10.42	7,794–8,168	7,981	7,981
	815	XA51840	CH	8,085	25	−30.9	8,987–9,086	9,013	9,016

*OM, organic matter; PF, plant fragment; CH, charcoal.

The core TJA consists of (1) stiff mud layer, overlying by yellowish clayey silt (714–550 cm), The average grain size is 3.57 μm, and the average contents of clay, silt, and sand are 43.2, 54.4, and 2.3%, respectively. (2) A grayish silty unit with OM and PF (550–399 cm). The particle grains become coarser from bottom to top as the silt content increases, with less contribution from clay. (3) Grayish clayey silt and then brownish silty clay with OM (399–220 cm). Please note, the depths 240–130 cm are correlated to the cultural layer of TJA. The average and median grain sizes are 5.59 μm and 8.29 μm, respectively, reaching the maximum value of the core, and the sand content increases gradually. (4) A section of peat layer occurs at 220–130 cm, followed by a section of brownish black clay (130–90 cm) with OM. Grayish and yellowish clay section with a few pieces of OM and Fe-Mn nodules (90–22 cm). It was then overlain by artificial fill at the top (22–0 cm). The median grain size and average grain size present a fine-coarse-fine change at the depth of 220–0 cm. The grain size of the peat layer is relatively fine. While the grain size of the brownish clay layer above the peat layer increases sharply, the sand content reaches a maximum of 6.9% in the entire core (Figure 2).

A brief description of sediment lithology of YJ1504 has been reported by Liu et al. (2018), and more details are given as follows. The basal unit of core YJ1504 contains (1) greenish stiff mud with Fe-Mn nodules at 900–820 cm. The average particle size is 19.59 μm, clay accounts for 21.03%, silt accounts for 74.89%, and sand accounts for 4.09%. (2) Coarse silty sediment with shell debris and gastropods at 820–490 cm. Notably, 770–490 cm is correlated to the cultural layer of JTS. The average grain size is 27.31 μm. Clay accounts for 18.98%, silt accounts for 70.35%, the proportion of the two components is lower than the lower unit; sand accounts for 10.67%, which is higher than the lower unit. (3) Finer grayish clay with a few silty laminae at 490–150 cm. The average grain size of this unit is the lowest among the four units, only 15.60 μm, with clay accounting for 27.45%, silt accounting for 69.37%, and sand accounting for 3.18%, the components of clay and silt being the lowest among the four units. (4) Dark grayish silty clay at the top (above 150 cm) interbedded with a peaty layer at 90–70 cm. The average grain size of this layer is 18.01 μm, with clay accounting for 23.85%, silt accounting for 72.41%, and sand accounting for 3.74% (Figure 3).

### Microfossil results

#### Phytoliths and foraminifera in TJA

**Figure 4:**
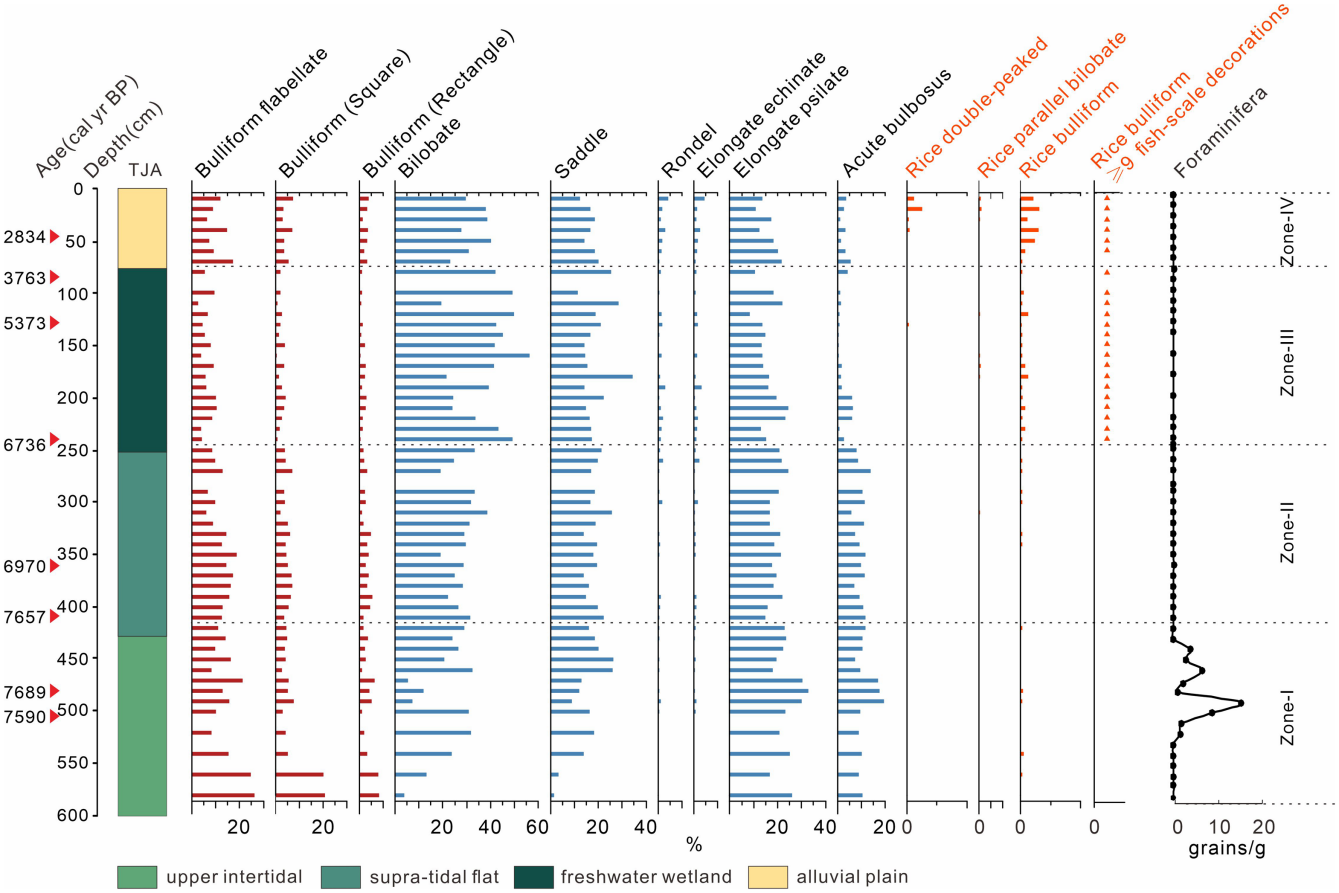
Assemblages of selected phytoliths and foraminifera of TJA.

A total of 31 morphotypes of phytoliths were identified from the TJA core. Most of them belong to the Poaceae family and major types were selected and presented in Figure 4. Bilobate, elongate psilate, bulliform, and saddle are the most common types in all samples. According to the changes of lithology and phytolith percentage, the phytolith assemblage can be divided into four zones.

Zone I (580–410 cm, *ca.* 7,810–7,350 cal yr. BP), high proportions of bulliform flabellate, bulliform, saddle, bilobate, elongate psilate, and acute bulbosus appeared, with lower percentages of rondel and elongate echinate phytoliths. Rice phytoliths first occurred at 560 cm (*ca.* 7,770 cal yr. BP), while the fish-scale decorations of rice bulliform were difficult to identify because of strong weathering. Foraminifera appeared only in this zone since 7,680 cal yr. BP (520 cm) and peaked at 7,620 cal yr. BP (490 cm), and then diminished at 7,460 cal yr. BP (440 cm).

Zone II (410–240 cm, *ca.* 7,350–6,600 cal yr. BP), bulliform flabellate, bulliform, saddle, elongate psilate, and acute bulbosus were still abundant. The proportions of the saddle and bilobate increased rapidly, while those of bulliform flabellate and bulliform, decreased slightly. Rice parallel bilobate first occurred at *ca.* 6,900 cal yr. BP (310 cm), and more importantly, rice bulliforms with ≥9 fish-scale decorations became visible at 6,600 cal yr. BP.

Zone III (240–80 cm, *ca.* 6,600–3,670 cal yr. BP), the proportions of bulliform flabellate, bulliform and acute bulbosus decreased obviously, while bilobate and elongate echinate presented an increasing trend. Rice bulliform and rice parallel bilobate increased markedly, with more appearances of bulliform >9 fish-scale decorations.

Zone IV (80–20 cm, *ca.* 3,670 cal yr. BP to the present), The proportions of bulliform flabellate, and bulliform acute bulbosus increased again, while bilobate and saddle exhibited a decreasing trend. The proportions of rice phytoliths increased to a peak of 3% at 20 cm.

#### Pollen, dinoflagellate, and charcoal in TJA

**Figure 5:**
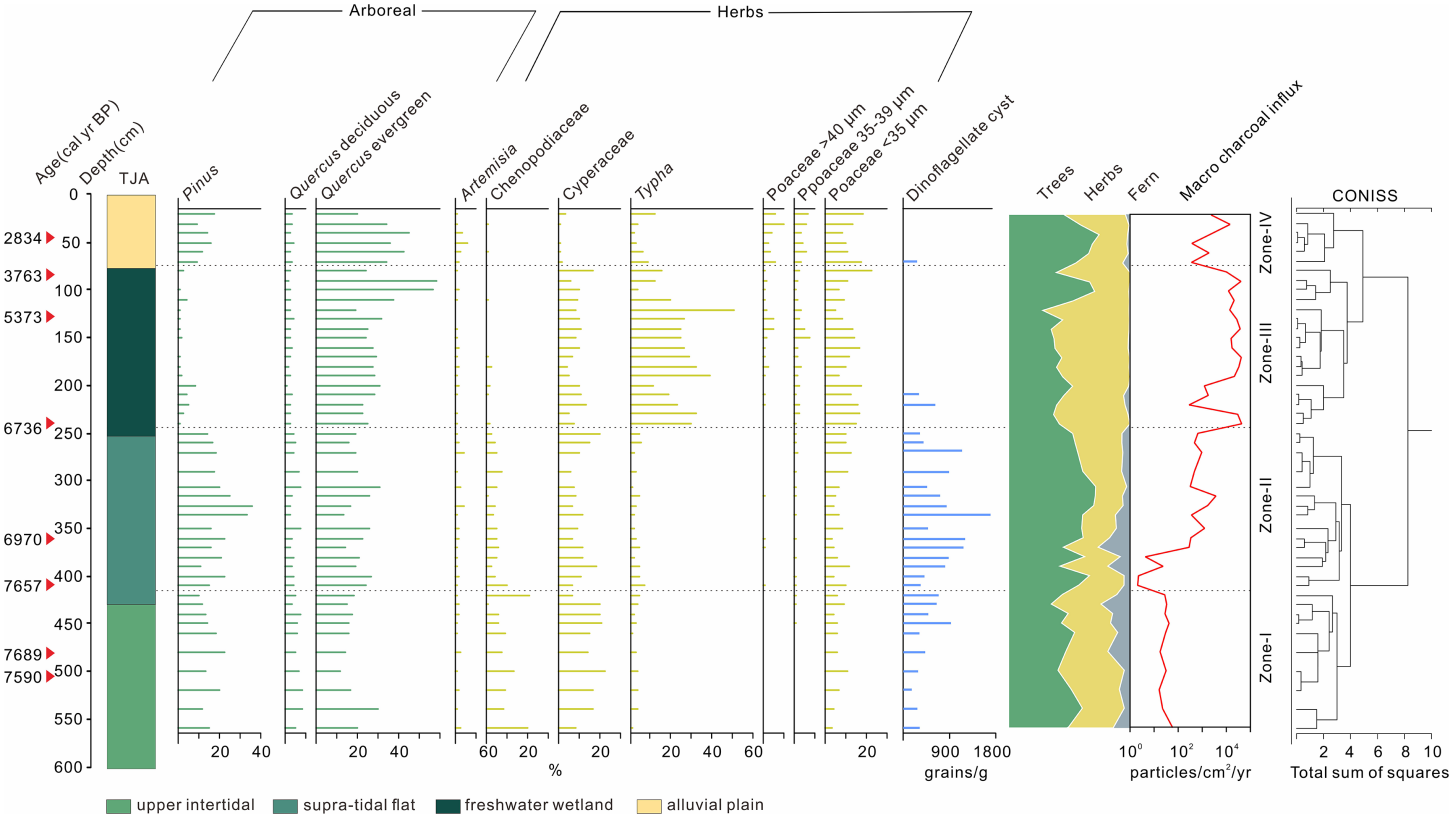
Assemblages of selected pollen species, concentration of dinoflagellates and variation of macro charcoal influx of TJA.

Sixty-five pollen-spore types were identified in the TJA core, including 35 arboreal taxa, 20 non-arboreal taxa, and 10 types of ferns. Four pollen-spore zones were classified by clustering analysis using Tilia, as shown in Figure 5.

Zone I (560–410 cm, *ca.* 7,770–7,350 cal yr. BP), high proportions of trees and shrubs were seen at this zone, including *Pinus*, *Liquidambar*, and evergreen and deciduous *Quercus*. The percentage of arboreal was 47.58%, while those of herbs were only 39.87%. Wetland indicators were mainly composed of Poaceae, *Typha*, Cyperaceae and Chenopodiaceae. And the percentage of Poaceae (>35 μm) was less than 1%. Dinoflagellate appeared continuously in this zone from bottom to top and the macro charcoal influx was generally below 10^2^ particles/cm^2^/year.

Zone II (410–240 cm, *ca.* 7,350–6,600 cal yr. BP), The percentages of *Pinus*, *Liquidambar*, evergreen *Quercus,* and fern types increased slightly. The percentages of Cyperaceae and Chenopodiaceae showed a small decrease, while the percentage of *Typha*, Poaceae (<35 μm), and Poaceae (35–39 μm) exhibited a decreasing trend. Poaceae (>40 μm) first appeared at 410 cm. The concentration of dinoflagellate increased and peaked at 7,010 cal yr. BP (340 cm). Notably, the macro charcoal influx showed an increasing trend from the bottom upward and approached 10^3^ particles/cm^2^/year.

Zone III (240–80 cm, *ca.* 6,600–3,670 cal yr. BP), the percentage of *Pinus*, deciduous *Quercus,* and fern types decreased distinctly. The content of evergreen *Quercus* rose to a high average of 29.81%. *Typha* showed a remarkable increase to 23.49%. Poaceae <35 μm, Poaceae 35–39 μm, and Poaceae >40 μm exhibited increasing trends. Dinoflagellate appeared only at 6,279–6,267 (220–210 cm), while the macro charcoal influx was kept high in this zone.

Zone IV (80–20 cm, *ca.* 3,670 cal yr. BP to the present), the percentage of *Pinus* increased to 11.26%. *Liquidambar*, evergreen *Quercus,* and deciduous *Quercus* kept a stable level of 3.63, 33.67, and 3%, respectively. *Typha* decreased remarkably to 7.19%. Cyperaceae and Chenopodiaceae almost disappeared in this zone. Poaceae (35–39 μm) and Poaceae (>40 μm) continued to rise, reaching peaks of 6.73 and 9.88%, respectively. Except for the presence at 3,500 cal yr. BP (70 cm), dinoflagellate almost disappeared. Additionally, the charcoal influx decreased in this zone (Figure 5).

#### Phytoliths and algae assemblages in YJ1504

**Figure 6:**
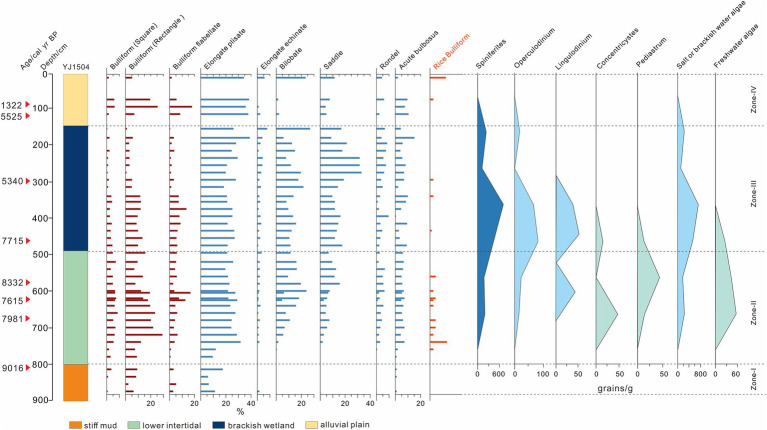
Assemblages of selected phytoliths and algae of YJ1504 to show the general trend of environmental change and related human activities.

A total of 32 morphotypes of phytoliths and 5 types of algae were identified from this core. Most phytolith types are similar to the core TJA and major types from the core YJ1504 were displayed in Figure 6.

Zone I (880–820 cm, late Pleistocene to 9,100 cal yr. BP): Bulliform flabellate, square, rectangular, and elongate plisate were common, and acute bulbosus appeared sporadically. The proportion of bilobate, saddle, and rondel phytoliths was relatively small and no rice-type phytoliths occurred. Algae identification was not applied to this zone.

Zone II (820–490 cm, *ca.* 9,100–7,120 cal yr. BP): The proportion of bulliform flabellate, square, rectangular, bilobate, saddle, and acute bulbosus all increased significantly. Rice bulliforms started to appear at 8,690 cal yr. BP (760 cm) and showed a continuous occurrence until 7,550 cal yr. BP (560 cm). Notably, the first trait of domesticated rice bulliform was observed at 7,830 cal yr. BP (620 cm). The algae community featured a mixture of saltwater and freshwater algae. The freshwater algae (*Concentricystes* and *Pediastrum*) reached its maximum at 7,980 cal yr. BP (650 cm) and started to decline afterward, and the saltwater algae (*Spiniferites*, *Operculodinium*, and *Lingulodinium*) showed an opposite trend from the bottom upward.

Zone III (490–150 cm, *ca.* 7,120–2,835 cal yr. BP): The bulliform types and the elongate types maintained their highs until 5,600 cal yr. BP (340 cm), after which the bulliform types started to decrease as the elongate types increased, as well as the saddle and bilobate. Rondel and acute bulbosus increased slightly since 5,600 cal yr. BP. Rice bulliform appeared occasionally at 6,610 cal yr. BP (435 cm), 5,600 cal yr. BP (340 cm) and 5,085 cal yr. BP (295 cm), among which the one at 435 cm showed the evident trait of domesticated rice, while the others could not be identified as domesticated rice due to severe weathering. The proportion of saltwater algae rose and started to dominate the algae community as freshwater algae gradually diminished. Saltwater species reached their maximum of at 5,700 cal yr. BP (350 cm) and then declined.

Zone IV (150 cm to the top, *ca.* 2,835 cal yr. BP to the present): The bulliform flabellate, square and rectangular showed a remarkable increasing trend in this zone. High values of elongate plisate occurred while the elongate echinate, bilobate, and saddle decreased. The rice bulliform phytoliths occurred at times, with the highest value seen at the top. The saltwater algae appeared in a low concentration and finally disappeared at 1,880 cal yr. BP (100 cm).

## Discussion

### Interpretation of environmental background for early settlements in the SHB

The Holocene environmental change of the SHB has been demonstrated by previous research, which provided a fundamental understanding of the stratigraphic evolution in response to the post-glacial sea-level change (Lin et al., 2005; Zhang et al., 2014; Liu et al., 2018, 2021). The depositional record of TJA and YJ1504 would have documented further details of environmental changes at specific locations with special implications for the establishment of early settlements.

A stiff mud layer, widely distributed in the Lower Yangtze Region, was recognized in both cores (Figures 2, 3). This typical deposition was formed in an exposure environment on the palaeo interfluves free of marine influence during the termination of the late Pleistocene (Li et al., 2002; Qin et al., 2008; Liu et al., 2020). To date, contemporaneous human activities were rarely reported in the coastal area, nor did our phytoliths evidence at YJ1504 would suggest so (Figure 6). Notably, the shallow burial depth of the stiff mud layer showed a higher palaeo relief in the early Holocene at both sites, which could act as a shelter in favor of early human occupation.

Above the stiff mud layer, the sediment showed a coarsening trend upward that suggested a strengthened local hydrodynamic condition (Figures 2, 3). The first appearance of saltwater algae mixed with freshwater species at 8,625 cal yr. BP in YJ1504 marked the beginning of marine influence (Figure 6). This was almost coeval with the occurrence of foraminifera in the nearby cores YJ1503 and YJ1505 (*ca.* 8,900 cal yr. BP), corresponding to the regional marine transgression processes (Dai et al., 2018). The occurrence of rice, which favored a brackish water setting (Qin et al., 2011), would further approve a tidal flat environment formed at the site during the early Holocene (Figure 6). On the contrary, a much later marine influence was identified at the TJA site, as evidenced by the belated occurrence of foraminifera and the increase of dinoflagellate at approx. 7,600 cal yr. BP, possibly because of its higher topography (Figure 4). In addition, the sporadic appearance of rice bulliform, and the well-matched co-occurrence of salt-tolerant herbs, such as Chenopodiaceae and Cyperaceae, would indicate an upper intertidal environment because of sea-level rise.

Notably, this marine influence at TJA was short-lived as implied by the disappearance of foraminifera at 7,350 cal yr. BP. Since then, a relatively stable environment was formed as reflected by fine grayish sediment and the frequent occurrences of OM. However, the persistent occurrence of Chenopodiaceae, Cyperaceae, and dinoflagellate would indicate a salty environment that remained at the site area (Figure 5). Compared to TJA, the persistent existence of saltwater algae informed a long-lasted impact from brackish water at YJ1504, which did not fade away until 5,700 cal yr. BP. This long-lasted marine influence was unique in the SHB, not even similar to the nearby locations such as YJ1503 and YJ1505 where the marine impact had ended at 7,600 cal yr. BP. We assumed that the locality of YJ1504 proximal to local tidal creeks would have favored the tidal force to bring saltwater algae into the YJV (Qin et al., 2011), especially when minor sea-level fluctuations still occurred in the SHB (He et al., 2018).

Since 6,600 cal yr. BP, the formation of a peaty layer at TJA and multiple locations in the SHB was informative of a stable environment created in the process of coastal progradation (Liu et al., 2018). The abundance of *Typha* and Poaceae pollen and the increasing proportion of domesticated rice bulliform phytoliths in the peaty layer indicated a freshwater setting. A decrease of *Pinus* and an increase of many anthropogenic indicators would imply an intensified human activity, possibly including forest opening, rice cultivation, and domestication at TJA (Figures 4, 5). At YJ1504, the reduction of saltwater algae at 5,700 cal yr. BP would indicate a gradually freshening environment due to a belated retreat of marine influence compared to TJA. In particular, occurrence of rice phytoliths at 5,600 cal yr. BP (340 cm) in YJ1504 might correspond to the amelioration of the regional environment as validated by previous findings from nearby regions (He et al., 2018, 2020b; Li et al., 2021).

In the late Holocene, the occurrence of yellowish sediment implied an oxidizing and drying condition. At TJA, the *Pinus* reclaimed its proportion at the expanse of *Quercus*. Although most salt-tolerant and aquatic herbs declined over time, *Artemisia* and Poaceae increased markedly, corresponding to the high values of rice bulliform phytoliths. By then, the microfossil evidence implied a transition from a wetland setting into a desiccated terrestrial environment. A similar condition could be inferred by the grayish silt deposit at YJ1504, within which algae diminished completely, indicating a desiccated terrestrial environment prevailed in the late Holocene.

Briefly, the regional sea-level pattern and marine transgression-regression sequence played a critical role in the evolution of the regional environment. An early start of marine influence at 8,625–7,980 cal yr. BP at YJ1504 was correlated to an estuary setting in the YJV following a marine transgression. As the sea-level gradually approached –5 m by 7,600 cal yr. BP, the marine invasion was at its maximum and had inundated many locations even with higher palaeo relief, including TJA. Since then, regional sea-level continued to rise but in a slow mode which allowed the initiation of coastal progradation. Consequently, the regional environment was gradually freshened and finally invoked the establishment of a freshwater setting at the TJA site and many other places since 6,600 cal yr. BP. However, the proximity of core YJ1504 to the tidal channel would be accountable for the late formation of freshwater wetland until 5,800 cal yr. BP, coeval to the widespread alluvial plain near the YJV as evidenced by the microfossil records from multiple locations (Liu et al., 2016; Tang et al., 2019b; He et al., 2020b; Li et al., 2021).

### Evolution of Neolithic subsistence near the core site

The transformation of subsistence strategies from hunting-gathering to farming was a revolutionary event in the history of humankind (Childe, 1936; Diamond, 2002). As known for the early birth of rice, the lower Yangtze region held essential information on the cultivation and domestication of rice for a comprehensive understanding of subsistence change over time (Zong et al., 2007; Fuller et al., 2009; Wu et al., 2014; Zuo et al., 2017; He et al., 2020a). Rice cultivation in Kuahuqiao area *ca.* 8,000 cal yr. BP was once considered as a sole event on the coastal wetlands of SHB. After a recent excavation of a contemporaneous Jingtoushan archaeological site (*ca.* 8,200–7,800 cal yr. BP) in the nearby YJV, new findings from microfossil and macrofossil evidence would indicate otherwise (Zhejiang Provincial Institute of Cultural Relics and Archaeology, Ningbo Institute of Cultural Heritage Management, Yuyao Hemudu Site Museum, 2021). The discovery of clams, oysters, saltwater fishes, and fishing tools proved the exploitation of marine resources by the local settlers. In addition, the unearthed plant remains of oaks, acorns, kiwifruits, and peaches suggested that abundant food resources were available for gathering near the Jingtoushan site. Phytolith records at YJ1504 near the Jingtoushan site showed the earliest occurrence of rice bulliform phytoliths at 760 cm (*ca.* 8,690 cal yr. BP) (Figure 6), though it did not show the evident trait of domesticated rice due to severe post-depositional weathering. At 620 cm (*ca.* 7,830 cal yr. BP), the first occurrence of cultivated rice (bulliform cell with more than nine fish-scale decorations) was believed coeval with the human occupation at the Jingtoushan site. Additionally, rice phytoliths of cultivated types were found at *ca.* 7,800–7,600 cal yr. BP in YJ1503 close to the Jingtoushan site, validating that the early settlers in the YJV area might have started rice cultivation no later than 7,800 years ago (Deng et al., 2021). At the meantime, macrofossil remains and microfossil evidence from the JTS site would indicate rice cultivation was performed by the local people throughout their occupation period (*ca.* 8,300–7,800 cal yr. BP) at the site (Zhejiang Provincial Institute of Cultural Relics and Archaeology, Ningbo Institute of Cultural Heritage Management, Yuyao Hemudu Site Museum, 2021; He et al., 2022). Moreover, archaeological findings from the JTS site suggested an upgrade of farming tools from shell and bone *Si* (耜) ploughs to wooden ploughs to facilitate rice cultivation (Zhejiang Provincial Institute of Cultural Relics and Archaeology, Ningbo Institute of Cultural Heritage Management, Yuyao Hemudu Site Museum, 2021). Thus, it seemed that the subsistence strategy of the Jingtoushan site might have been a mixture of primitive agriculture and hunting-gathering.

**Figure 7:**
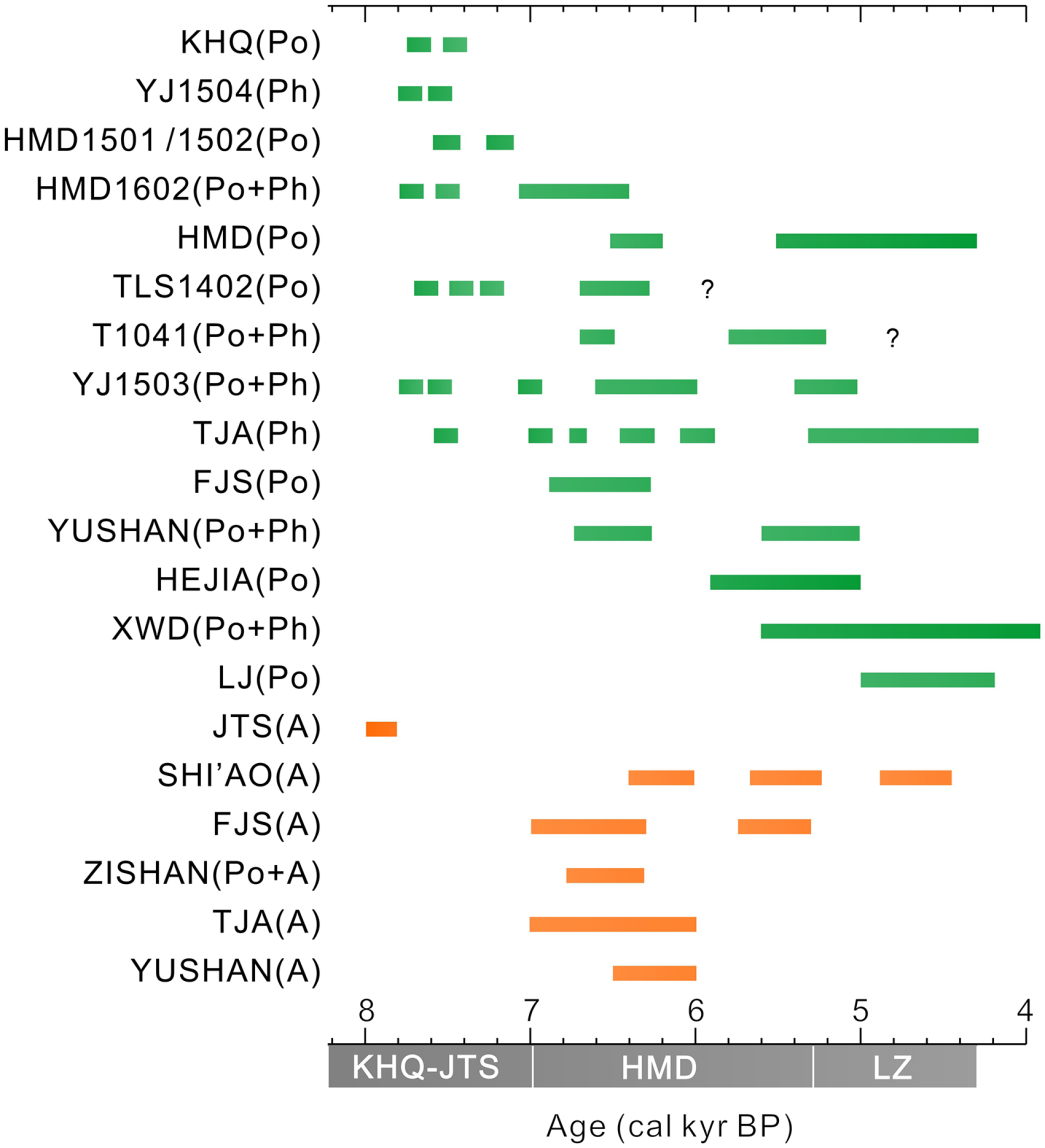
Collected information on the rice planting history (including cultivation and domestication) in the YJV. The horizontal axis represents the age of the occurrence of rice planting revealed in different sediment cores or archaeological sites. The vertical axis represents the cores or sites with rice planting history. “Po” represents evidence of rice planting supported by pollen records. “Ph” represents phytolith evidence and “A” represents archaeological findings. “JTS” represents JTS occupation. “KHQ” represents Kuahuqiao Culture. “HMD” represents Hemudu Culture. “LZ” represents Liangzhu Culture (The relevant data came from Wang and Zhang, 2001; Zhejiang Provincial Institute of Cultural Relics and Archaeology, Department of History Xiamen University, 2001; Zong et al., 2007; Archaeological Research Center of Peking University and Zhejiang Provincial Institute of Cultural Relics and Archaeology, 2011; Qin et al., 2011; Li et al., 2012, 2021; Ningbo Municipal Institute of Cultural Relics and Archaeology, 2013; Ningbo Municipal Institute of Cultural Relics and Archaeology, Cixi Museum, 2013; Liu et al., 2016, 2020; Zheng et al., 2016; He et al., 2018, 2020a,b; Ma et al., 2018; Tang, 2019; Deng et al., 2021; Zhejiang Provincial Institute of Cultural Relics and Archaeology, 2021).

Nevertheless, although rice usage started early at JTS, the subsequent domestication might be discontinuous. Our phytoliths records at YJ1504 and other published data from sediment cores and archaeological sites showed intermittent rice cultivation appeared between 7,800–6,600 cal yr. BP in the YJV (Figure 7). Moreover, microfossil evidence from TJA beyond the YJV showed occasional occurrences of Poaceae pollen (>35 μm) and rice bulliform during 7,800–6,600 cal yr. BP, although without clear signatures of domestication (Figures 4, 5). Despite that, the abundant *Pinus*, *Quercus*, and a small amount of *Typha* in the pollen assemblages might signify the availability of edible resources such as acorns, oaks, pine cones, and cattails, commonly collected by local people (Fuller and Qin, 2010; Pan and Yuan, 2018; Zhang et al., 2020). The increasing trend of macro-charcoal influx would also suggest a gradually intensified human activity around the TJA site during 7,800–6,600 cal yr. BP (Figure 5). Since then, domesticated rice phytoliths occurred at 240 cm demonstrated that local rice cultivation started at 6,600 cal yr. BP, co-occurring with many other sites in the region, such as Tianluoshan, Hemudu, and Yushan (Figures 4, 7). During 6,600–3,700 cal yr. BP, Poaceae pollen (>35 μm), rice bulliform phytolith, and the domesticated types showed a continuous and increasing pattern, indicating that rice domestication was a persistent practice at the TJA site. This assumption was supported by multiple farming *Si* (耜) ploughs made of animal bone unearthed from the cultural layers T1-5 and T2-8 of the TJA site, corresponding to *ca.* 7,000–6,300 cal yr. BP (Ningbo Municipal Institute of Cultural Relics and Archaeology, Cixi Museum, 2012). In addition, the increasing content of *Quercus* and *Typha* would imply that abundant wetland resources and acorns were available, attracting more Neolithic settlers and leading to a persistent occupation at the site, which included the construction of a paved road in the late Hemudu Culture period (5,700–5,300 cal yr. BP).

### Subsistence changes and the corresponding environment in the SHB

**Figure 8:**
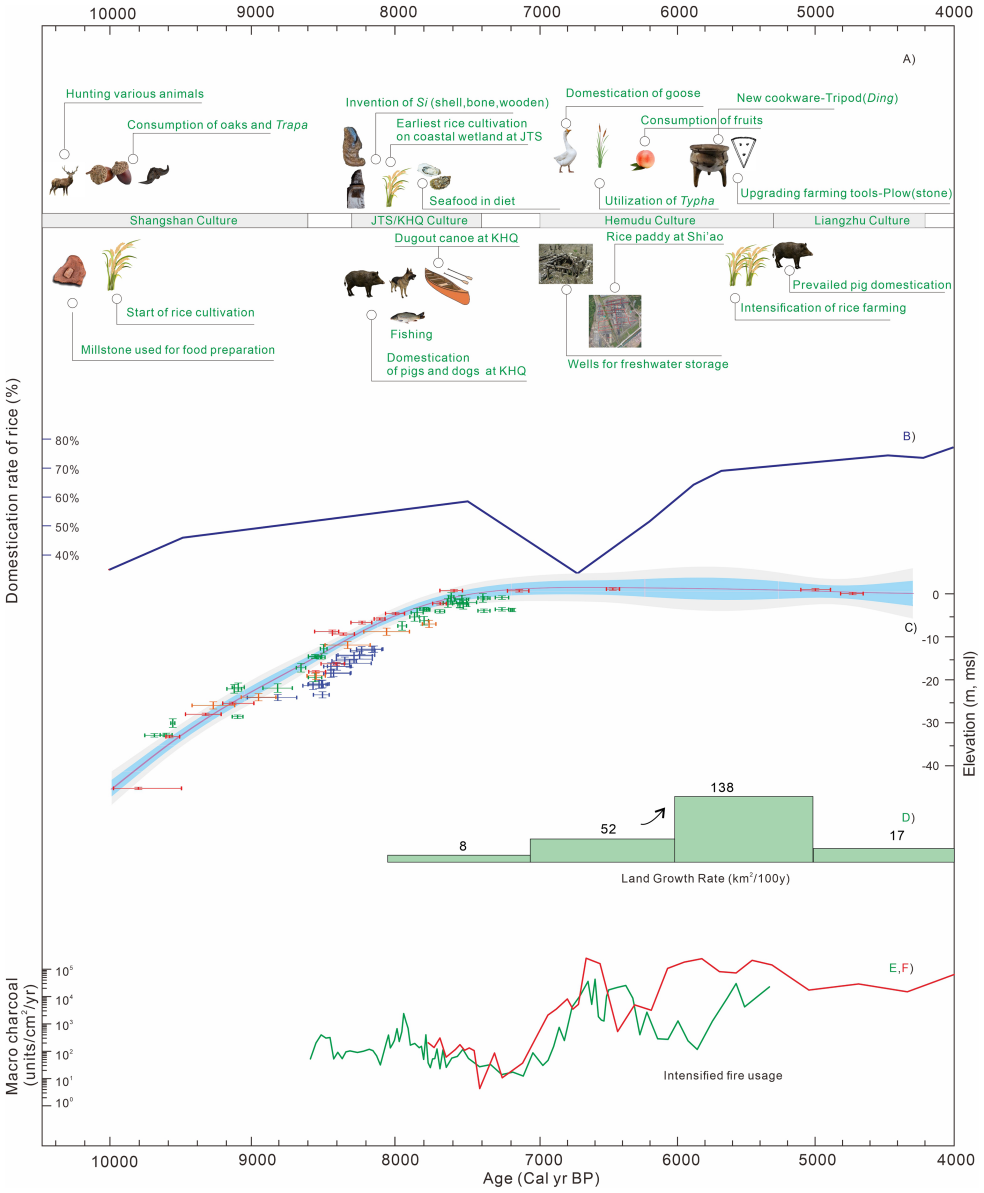
**(A)** Synthesis of the transformation process of subsistence strategies and landscape changes in the SHB. At each cultural stage, the most representative features of subsistence strategy were marked in the figure (data collected from Zong et al., 2007; Archaeological Research Center of Peking University and Zhejiang Provincial Institute of Cultural Relics and Archaeology, 2011; Jiang, 2014; Zhejiang Provincial Institute of Cultural Relics and Archaeology, 2016, 2021; Zuo et al., 2017; Zhang et al., 2020; Eda et al., 2022; Official account of Qianjiang Evening News, 2022). **(B)** Trends of the proportions of domesticated-type bulliform phytoliths from rice (Ma et al., 2016). **(C)** Holocene sea-level changes in the Hangzhou Bay (Xiong et al., 2020). **(D)** Land growth rate of SHB since coastal progradation at ~8,000 cal yr. BP (Liu et al., 2021). **(E)** Macro-charcoal influx of YJ1503 at JTS site (Liu et al., 2020). **(F)** Macro-charcoal influx of TJA site.

Since the middle Holocene, the worldwide initiation of the coastal plain had provided arable land and extra wetland resources for ancient settlers and catalyzed the subsequent development of the agricultural economy in many coastal regions. In the SHB, the coastal plain initiation since 8,000 cal yr. BP was attractive to Neolithic settlers, whose subsistence evolved from a broad spectrum of food resources to intensive agriculture as the Neolithic culture developed from individual settlements to a hierarchical society (Figure 8).

In general, the cold climate of the Late Glacial Maximum was not supportive of a significant human occupation nor a dense distribution of wild rice in the open area of SHB (Lu, 1999). To date, the absence of phytoliths or Poaceae pollen (>40 μm) and low concentration of charcoal in the stiff mud layer indicated rare human activity in the YJV before the Holocene (Figure 7; Liu et al., 2020). On the contrary, the warming climate in the Holocene boosted the cultivation and domestication of various crops (Larson et al., 2014). As one of the most popular food at present, rice was considered the first plant cultivated by the Neolithic people of the Shangshan Culture under the co-occurring strengthening monsoon climate (Wu et al., 2014; Zuo et al., 2017; Figure 8A). Phytoliths evidence indicated that rice domestication at that time was still at an introductory level, and rice was a supplementary food choice in addition to a large variety of natural food resources (Figure 8B; Zhao and Jiang, 2016). However, recent research claimed that higher rates of rice domestication usually occurred at those sites close to the mainstream river (Huan et al., 2021), implying that the local environment could serve as a critical factor in the process of rice domestication. In addition, starch remains from the Qiaotou site showed that ancient people might have used rice for beer-brewing for a ritual purpose (Wang et al., 2021).

As the rate of sea-level rise slowed down at ~8,000 cal yr. BP, coastal wetland started to initiate at locations with semi-enclosure geometry (Figure 8C; Liu et al., 2021), such as the Kuahuqiao and Yaojiang Valley (Jingtoushan site). Diatom and pollen evidence indicated a freshwater environment at the Kuahuqiao site (Zong et al., 2007; Wu et al., 2016) and a brackish wetland setting in the YJV provided material bases for human settlements (Supplementary Table S1). Charcoal analysis from both sites indicated that local people had adopted a slash-and-burn strategy to open vegetation for more resources and arable land (Hu et al., 2020; He et al., 2022). Rice cultivation was confirmed at both sites assisted by primitive farming tools (Figure 8A). In the meanwhile, high contents of aquatic fungi, macrophytes, and algae may imply the existence of local swamps with open water bodies (Innes et al., 2009; Liu et al., 2020), which provided fish, *Typha*, and water chestnuts to the ancient people for daily subsistence. The unearthed animal bones of buffalo and deer, together with the remains of acorns, oaks, and peaches, reflected the intake of terrestrial resources by the local people, as evidenced by the results of C and N isotopes from human bones (Hu, 2021). In addition, we might infer a preference for seafood for the Jingtoushan people compared to the Kuahuqiao people based on the abundant oyster shells found at the Jingtoushan site. Above all, such a combination of food supply indicated a dominant hunting-gathering economy supplemented with rice cultivation at Kuahuqiao and Jingtoushan sites.

To date, no other archaeological sites of *ca.* 8,000 cal yr. BP were reported in the coastal plains of SHB except for the Kuahuqiao and Jingtoushan sites. However, human occupations at both locations were interrupted by unstable hydrological conditions at 7,600–7,000 cal yr. BP. It remained debatable whether sea-level rise or local tidal force was accountable for the discontinued human occupation at either site (Zong et al., 2007; Liu et al., 2020). Previous studies held a theory that human activities were absent during 7,600–7,000 cal yr. BP due to the lack of contemporaneous archaeological findings. Nevertheless, recent paleontological studies on sediment cores obtained from Jingtoushan, Tianluoshan, and Hemudu sites suggested that there could be intermittent human activities and rice cultivation in the YJV during 7,600–7,000 cal yr. BP (Figure 7; Supplementary Figures S1, S2; Ma et al., 2018; He et al., 2020b; Deng et al., 2021). The locality of these sites was close to foothills with relatively higher palaeo relief that were less affected by the Holocene marine invasion. Such intermittent human activities appeared at a time when the marine transgression gradually transited to regression, allowing the process of coastal progradation with an increasing rate of land growth, which allowed development of a vast alluvial plain with a progressively freshening environment (Figure 8D; Liu et al., 2018).

As the coastal progradation continued after 7,000 cal yr. BP, more sites were occupied by the Neolithic people during the Hemudu cultural period. In addition to the previously occupied Jingtoushan, Tianluoshan, and Hemudu sites, new settlements were established at locations such as Zishan, Xiangjiashan, Fujiashan, and Tongjia’ao and Yushan since 7,000–6,600 cal yr. BP (Supplementary Figure S3). Noteworthy, all of these locations were close to foothills with freshening wetland as suggested by lithological and palynological evidence, indicating a preference for site selection for the settlers during the early stage of the Hemudu culture. Since the formation of freshwater wetland, intense human activities were indicated by the high values of macro-charcoal influx, as represented by the JTS and TJA sites (Figures 8E,F). In addition, rice cultivation occurred at all these locations as evidenced by macro remains and microfossil evidence of rice, or relics of farming tools (Figure 7). Moreover, a large rice paddy recently found at Shi’ao dated back to 6,700 cal yr. BP was interpreted to be 8 ha in size (Figure 8A). This rice paddy was the largest ancient rice paddy in the lower Yangtze region by now, indicating significant labor input and developed technology for rice farming during the Hemudu cultural period, which could be acknowledged as a human adaptation to and utilization of the wetland environment. However, the rice yield at that time was predicted of just 830 kg/hectare which might not meet the daily need (Zheng et al., 2009). We assumed that such preliminary rice productivity may not sustain the development of the local society unless a stable hydrological condition was provided. In addition, as indicated by previous findings, the production rate (calorie/h) of rice was significantly lower than natural resources (Lu, 1999). Although rice cultivation became widely practiced in the Hemudu cultural period, it could only contribute a minor proportion of daily energy intake. Most of the energy intake still came from natural resources, including at least 58 species of wild animals and a large number of terrestrial nuts and fruits (Zhejiang Provincial Institute of Cultural Relics and Archaeology, 2003; Figure 8A). Such a subsistence style developed at multiple locations of SHB as the coastal progradation continued. Although human occupation and rice domestication were occasionally affected by unstable hydrological processes (He et al., 2018; Liu et al., 2020; Long, 2021), the rate of rice domestication increased evidently (Figure 8B). This was highly likely associated with the increasing demographic pressure, and the advances in farming technology (Zheng et al., 2009, 2012; Deng et al., 2021; Huan et al., 2021).

Since 5,500 cal yr. BP, rice farming has become a common practice at various sites in SHB (Figure 7), and many locations on the north flank of Hangzhou Bay, supporting the significantly increased population. This process was coeval with the rapid land growth and formation of vast alluvial plains when the shoreline had prograded northward and eastward in SHB (Figure 8D; Liu et al., 2021). The invention of the stone plough during the last period of Hemudu Culture (which co-occurred with Songze Culture in the north Hangzhou Bay) greatly improved farming productivity and domestication rate of rice (Figure 8A) and intrigued the prevalence of rice farming across the lower Yangtze region. By then, rice started to serve as one of the main staple foods in the lower Yangtze region to provide carbohydrates. Finally, it took a long time of over 5 millennia for the strategy of ancient human subsistence to shift from reliance on hunting-gathering to a farming economy in the lower Yangtze region, which had a prolonged influence in the history of China’s civilizations and is still adopted by Chinese people in modern time.

## Conclusion

The Holocene environmental changes at two archaeological sites (JTS and TJA) generally corresponded to the regional sea-level fluctuation pattern. Yet, local topography and landform also played a critical role in the environmental processes. The transition of Neolithic subsistence was affected by complex factors such as demographic pressure, technological advances, and societal development, and the present study highlighted the role of environmental change. Our results showed that the initial wetland formation in the semi-enclosure environment at Kuahuqiao and Yaojiang Valley of SHB had enabled the activities of hunting, gathering, and fishing *ca.* 8,000 years ago. Rice cultivation also provided a supplementary food source. Since 7,600 cal yr. BP, the differentiated process of wetland desalinization in different areas showed varied responses to the regional sea-level pattern. Records from sediment cores at multiple sites provided new information on intermittent rice cultivation within the gradually freshened wetland. During 7,000–6,600 cal yr. BP, a wide variety of natural food resources still dominated the daily diet of the Neolithic people. However, as freshwater wetlands expanded in the process of coastal progradation, rice cultivation became widely practiced. After 5,500 cal yr. BP, as the shoreline migrated further seawards and the alluvial plain developed, a stable freshwater environment was formed that ensured a suitable hydrological condition for rice to grow. In the meanwhile, advances in farming tools and paddy management facilitated rice farming and made rice a common choice in the diet. From hunting-gathering to rice farming, the subsistence changes showed an adaptive strategy in response to landscape evolution from an initial marine-influenced setting to a later coastal plain environment. The final establishment of an agricultural subsistence eventually formed the economic foundation of the prosperous civilization of China.

## Data availability statement

The raw data supporting the conclusions of this article will be made available by the authors, without undue reservation.

## Author contributions

JD, YL, and QS conceptualized the study and wrote the manuscript. JD, LD, DF and XSZ implemented the experiment and collected the data. LD and DF performed the statistical analysis. JD, QS, ML, JC, ZC, and YL wrote and edited the manuscript, JD, QS, YL, HW, LX, XL, XYZ revised the manuscript and redrawn the figures. QS and YL acquired funding. All authors contributed to the article and approved the submitted and final version.

## Funding

This research and the OA publication were funded by the National Natural Science Foundation of China (Grant Nos. 42071110 and 41971007).

## Conflict of interest

The authors declare that the research was conducted in the absence of any commercial or financial relationships that could be construed as a potential conflict of interest.

## Publisher’s note

All claims expressed in this article are solely those of the authors and do not necessarily represent those of their affiliated organizations, or those of the publisher, the editors and the reviewers. Any product that may be evaluated in this article, or claim that may be made by its manufacturer, is not guaranteed or endorsed by the publisher.
