# Supplementary material for: Changes of Neolithic subsistence in south Hangzhou Bay coast, eastern China: An adaptive strategy to landscape processes
Source: Front Plant Sci. 2022 Sep 20;13:1000583. doi: 10.3389/fpls.2022.1000583 (PMC9531130; doi:10.3389/fpls.2022.1000583)
Supplement: Supplementary file 1 [file Data_Sheet_1.docx]

SUPPLEMENTARY MATERIAL

**Supplementary Table S1** *Integration of food sources information in representative archaeological sites in the lower Yangtze River*

| **Neolithic sites** | **Period** | **Food sources** | **References** |  |
| --- | --- | --- | --- | --- |
| Shangshan | *ca.* 11400–8600 BP | deer, bony fish, pigs, birds;  acorns, rhizomes, Job's tears, water chestnuts, rice, barnyard grass. | Zhejiang Provincial Institute of Cultural Relics and Archaeology, 2016 |  |
| Xiaohuangshan | *ca.* 9500–7000 BP | cattles; tubers, nuts, rice | Zhejiang Provincial Institute of Cultural Relics and Archaeology, 2016 |  |
| Qiaotou | *ca.* 9000 BP | Job's tears (*Coix lacryma-jobi*), rice, acorns | Zhejiang Provincial Institute of Cultural Relics and Archaeology, 2016; Wang et al., 2021 |  |
| Kuahuqiao | *ca.*8000–7250 BP | swimming crab (Portunidae), carp, northern snakehead and unknown fish, oyster, sinonovacula, turtle, dolphin, Yangtze crocodile; swan, duck, eagle, unknown birds;  mice, raccoon dog, dog, badger, ocelot, rhino, pig, elk, sika deer, small deer, buffalo and serow; peach, apricot, acorn, water chestnut (*Trapa*), gorgon fruit, Ziziphus jujuba (*Choerospondias axillaris*); plum (*Prunus mume*), acorns (*Quercus acutissima*, *Quercus variabilis*, *Quercus fabri*), gorgon fruit (*Euryale ferox*); bean (Leguminosae),gourd (*Cucurbitaceae*), persimmon (*Diospyros*), pondweed (Potamogetonaceae), tubers, rice | Jiang, 2014 |  |
| Xiasun | Late Kuahuqiao Culture | buffalo, domestic dog, domestic pig; carp, Huang sang fish (*Tachysurus fulvidraco*), swimming crab (Portunidae), oysters water chestnut (*Trapa*), rice | Jiang, 2014 |  |
| Jingtoushan | *ca.* 8300–7800 BP | oysters, clams, razor clam (*Sinonovacula* *constricta*), conch, cockles (*Tegillarca* *granosa*), tuna, cracorner, shark, ray; deer, dog, wild boar, buffalo; acorn (*Quercus* *acutissima*), kiwifruit (*Actinidia*), peach, diospyros, ganoderma, rice | Zhejiang Provincial Institute of Cultural Relics and Archaeology et al., 2021 |  |
| Hemudu | Phase 1 (*ca.* 7000–6500 BP) | Asian Elephant, Elephant, rhinoceros, monkey, tortoise, domestic pig, wild boar, dog, buffalo, roe (*Hydropotes inermis*), deers, muntjac, sambar, elk, sumatran serow (*Capricornis sumatraensis*), sika deer, porcupines, badger, raccoon dog, rhinocero, tiger, jackal, leopard cat, civet cat, ferret, crab-eating mongoose, pig badger (*Arctonyx collaris*), pangolins, black rats, macaques, black bear, pelican (Pelecanidae), cormorant (Phalacrocoracidae), crane, heron (Ardeidae), duck, goose, crow, eagle; anodon (*Anodonta*),swimming crabs (Portunidae), spiral shell, otter, smooth-coated otter (*Lutra perspicillata*); sea turtles, yellow-margined box turtles, tortoises, Chinese box turtle , whale, shark; sturgeon, carp, crucian (*Carassius auratus*), herring, *Gymnocranius griseus*, Huang sang fish (*Tachysurus fulvidraco*), bighead carp (*Hypophthalmichthys nobilis*), catfish, Flathead grey mullet (*Mugil cephalus*), snakeheaded fish (*Channa argus*); acorn, water chestnut, gorgon fruit, Ziziphus jujuba (*Choerospondias axillaris*), gourd, locust tree fruit (*Styphnolobium japonicum*), Job's tears (*Coix lacryma-jobi*), *Typha*, potamogetonaceae, lotus, red bayberry (*Myrica rubra*), peach, rice | Zhejiang Provincial Institute of Cultural Relics and Archaeology, 2003 |  |
|  | Phase 2 (*ca.* 6500–6300 BP) | poorly preserved |  |  |
|  | Phase 3 (*ca.* 6300–5700 BP) | poorly preserved |  |  |
|  | Phase 4 (*ca.* 5700–5300 BP) | poorly preserved |  |  |
| Tianluoshan | Under 8 layer (K3⑦) | pig, buffalo, sambar, sika deer, muntjac, badger, cat, otter; culter, cyprinus, carassius, blotched snakehead (*Channa maculata*); acorn, water chestnut, peach, Ziziphus jujuba (*Choerospondias axillaris*), gorgon fruit (*Euryale ferox*), diospyros, pine cone, gourd (Cucurbitaceae), rice | Archaeological Research Center of Peking University and Zhejiang Provincial Institute of Cultural Relics and Archaeology, 2011 |  |
|  | Layer 7, 8 (early cultural layers) *ca.* 7000–6500 BP | pig, buffalo, sambar, sika deer, muntjac, monkey, badger, wild cat, raccoon dog, bear; fish; water chestnut, acorn, pine cone, plum, gorgon fruit (*Euryale ferox*), diospyros, Actinidia, rubus, Ziziphus jujuba (*Choerospondias axillaris*), waxberry (*Myrica rubra*), grape, *Nymphoides*, lotus, rice |  |  |
|  |  |  |  |  |
|  | Layer 5,6 (mid cultural layers) *ca.* 6500–6000 BP | freshwater fish, tuna, sharks; ferret, raccoon dog, badger, wild cat, monkey, dog, porcupine, buffalo, elk, sambar, sika deer, roe deer, deer, muntjac, otter, bear; water chestnut, acorn, peach, Ziziphus jujuba (*Choerospondias axillaris*), Euryale ferox, diospyros, kiwifruit (*Actinidia*), *Rubus*, gourd (Cucurbitaceae), melon (*Cucumis melo*), *Typha*, rice |  |  |
|  |  |  |  |  |
|  | Layer 3,4 (late cultural layers) *ca.* 6000–5500 BP | pig, buffalo, deer, monkey, leopard, otter, badger, cat; peach, rice, acorn, water chestnut |  |  |
|  |  |  |  |  |
| Shi’ao | Hemudu Culture Phase 1 (*ca.* 4800–4500 BC) | rice paddy field | Official account of Qianjiang Evening News, 2022 |  |
|  | Hemudu Culture Phase 4 (*ca.* 3700–3300BC) |  |  |  |
|  | Liangzhu Culture (*ca.* 3300–2600 BC) |  |  |  |
| Zishan | Early Hemudu Culture (7000–6000 BP) | cartilaginous fish, pig, sika deer, muntjac, raccoon dog, turtle (*Pelochelys*), Chinese soft-shelled turtle, shark; acorns (*Quercus acutissima*), gourd (Cucurbitaceae), rice | Zhejiang Provincial Institute of Cultural Relics and Archaeology and department of History, Xiamen University, 2001 |  |
| Tongjia'ao | Phase 1 (Hemudu Culture Phase 1) | animals, rice | Ningbo Municipal Institute of Cultural Relics and Archaeology and Cixi Museum, 2012 |  |
|  | Phase 2 (Hemudu Culture Phase 2) |  |  |  |
|  | Phase 3 (Hemudu Culture Phase 3) |  |  |  |
|  | Phase 4 (Hemudu Culture Phase 4) |  |  |  |
| Xiangjiashan | Phase 1 (Hemudu Culture Phase 2) | deer, tortoise, acorns | Sun and Huang, 2000 |  |
|  | Phase 2 (Hemudu Culture Phase 3-4) |  |  |  |
|  | Phase 3 (Hemudu Culture Phase 4) |  |  |  |
|  | Phase 4 (The Spring and Autumn Period and the Warring States Period) |  |  |  |
| Fujiashan | Layer 8 (*ca.* 7000–6360 BP) | snakeheaded fish (*Channa argus*), Order Perciformes or salmonformes; Spoonbill, hu (鹱), tortoise, macaque, badger, otter, undetermined feline, rhinoceros, domestic pig, sika deer, sambar, muntjac, water buffalo; water chestnut (*Trapa*), acorns, Ziziphus jujuba (*Choerospondias axillaris*), Pinus, pondweed (Potamogetonaceae), Nymphoides, Citrus hybrid, pecan (*Carya*), walnut (*Juglans*), kiwifruit (*Actinidia*), diospyros, hazel (*Corylus*); rice | Ningbo Municipal Institute of Cultural Relics and Archaeology, 2013 |  |
|  | Layer 6,7 (*ca.* 6300–6000 BP) |  |  |  |
|  |  |  |  |  |
|  | Layer 3,4 (*ca.* 5700–5300 BP) |  |  |  |
|  |  |  |  |  |
| Cihu | Hemudu Culture (*ca.* 5747±110 BP) | Choerospondias axillaris, acorns (*Quercus acutissima*), peach, rice | Pan and Yuan, 2018 |  |
|  | Liangzhu Culture (*ca.* 5365±125 BP) |  |  |  |
|  | Layer 3 (Hemudu Culture Phase 4) |  |  |  |
|  | Layer 2 (Late Liangzhu Culture) |  |  |  |
| Yushan | Hemudu Culture Phase 2,3 (6800–6000 BP) | buffalo, sika deer, wild boar, tortoise and tuna;  water chestnut, gorgon fruit (*Euryale ferox*), acorn, melon (*Cucumis*), rice | Lei et al., 2016; Lei and Wang, 2016 |  |
|  | Liangzhu Culture (5100–4800 BP) | water chestnut, gorgon fruit (*Euryale ferox*), acorn, melon (*Cucumis*), rice |  |  |
| Wuguishan | Hemudu Culture Phase 3,4 | snakeheaded fish (*Channa argus*), carp (*Cyprinus carpio*), catfish (*Silurus*); mullet (*Mugil cephalus*), perch (*Lateolabrax japonicus*), Acanthopagrus;  Selachomorpha, Myliobatidae, Sciaenidae | Lei and Wang, 2016;  Zhu et al., 2020 |  |
|  | Liangzhu Culture |  |  |  |
| Xiawangdu | Phase 1 (Hemudu Culture Phase 4) | deer, pig, *Typha*, rice | Ningbo Municipal Institute of Cultural Relics and Archaeology et al., 2019 |  |
|  | Phase 2 (Liangzhu Culture) |  |  |  |
|  |  |  |  |  |
| Hejia | Phase 1 (Hemudu Culture Phase 4) | acorns, peach, rice | Ningbo Municipal Institute of Cultural Relics and Archaeology et al., 2019 |  |
|  | Phase 2 (early Liangzhu Culture) |  |  |  |
|  | Phase 2 (late Liangzhu Culture to Qianshanyang Culture) |  |  |  |
| Tashan | Hemudu Culture Phase 3 | freshwater fish, domestic pig, muntjac, small deer, sambar deer, sika deer, buffalo; pondweed (Potamogetonaceae), *Typha*, walnut (*Juglans*), persimmon (*Diospyros*), elm, rice | Zhejiang Provincial Institute of Cultural Relics and Archaeology and Xiangshan County Cultural Relics Management Committee, 2014 |  |
|  | Hemudu Culture, Songze Culture |  |  |  |
|  | Late Liangzhu Culture |  |  |  |





**Supplementary Figure S1** *Microfossil evidence related to rice domestication processes and human activities in the SHB before 7600 BP. BMH-Baimahu, KHQ-Kuahuqiao, JTS-Jingtoushan. (Wu et al., 2016; Hu et al., 2020; Liu et al., 2020; Deng et al., 2021; He et al., 2022)*


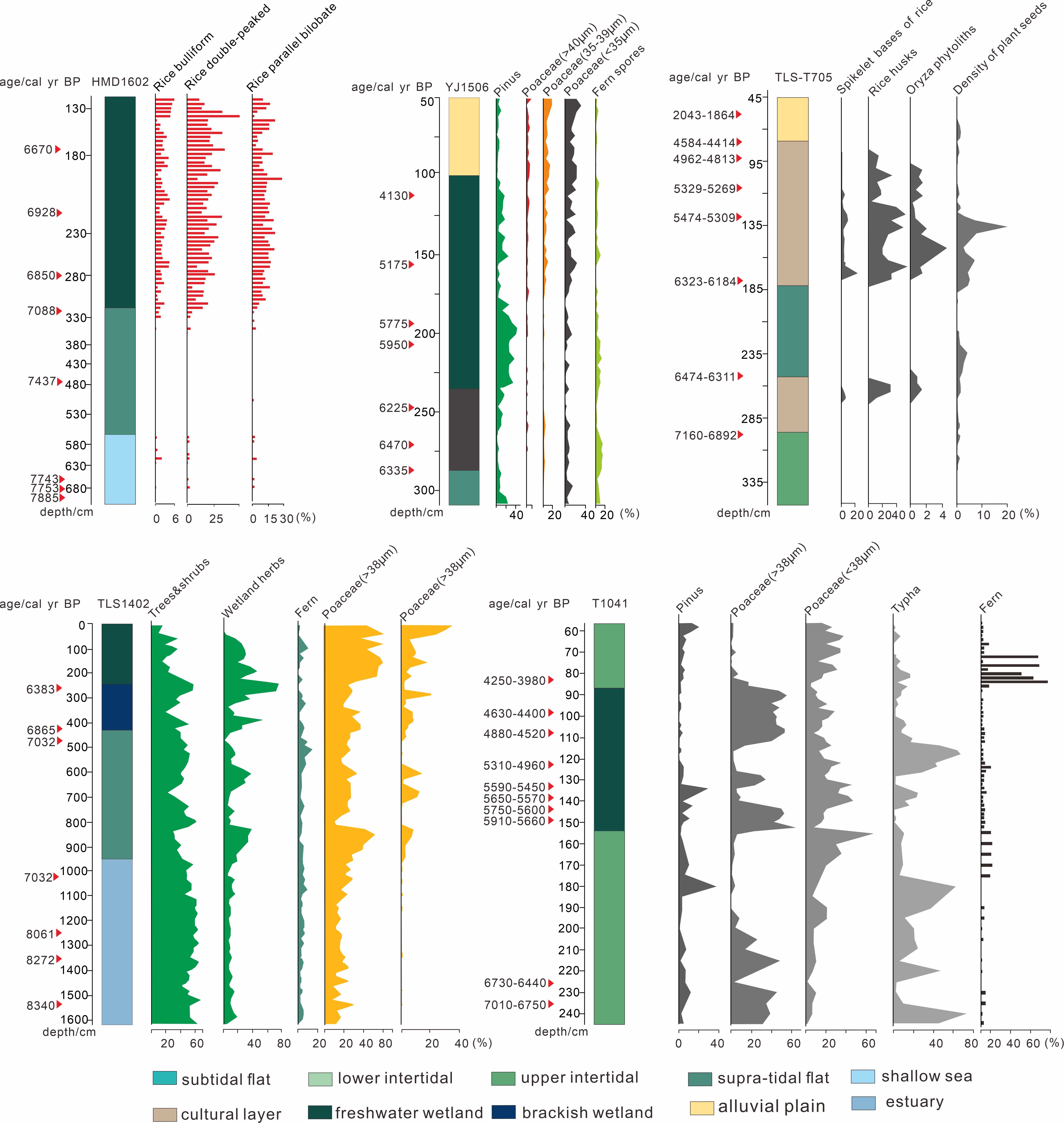


**Supplementary Figure S2** *Microfossil evidence related to rice domestication processes and human activities in the SHB during 7600–7000 BP. HMD-Hemudu, TLS-Tianluoshan. (Zheng et al., 2009; Li et al., 2012; Liu et al., 2016; Ma et al., 2018; He et al., 2020)*


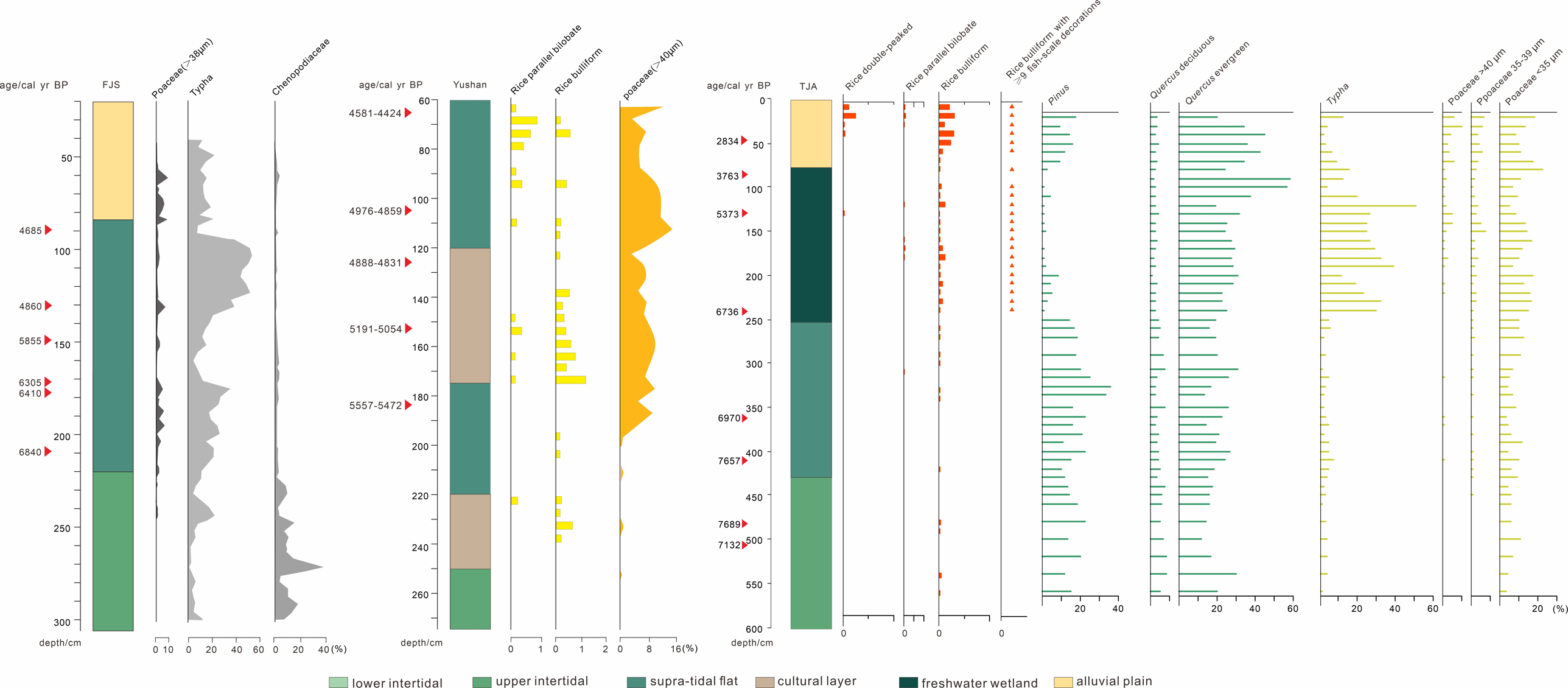


**Supplementary Figure S3** *Microfossil evidence related to rice domestication processes and human activities in the SHB after 7000 BP. FJS-Fujiashan, TJA-Tongjia’ao. (He et al., 2018; Tang, 2019)*

**References**

Archaeological Research Center of Peking University, Zhejiang Provincial Institute of Cultural Relics and Archaeology (2011). *A comprehensive study on the natural remains of the Tianluoshan site*. Beijing: Cultural relics publishing house. (in Chinese)

Deng L., Liu Y., He J., Jiang R., Jiang F., Chen J., Chen Z., Sun Q. (2021). New archaeobotanical evidence reveals synchronous rice domestication 7600 years ago on south Hangzhou Bay coast, eastern China. *Anthropocene*. 33. doi: 10.1016/j.ancene.2021.100280

He K., Lu H., Sun G., Wang Y., Zheng Y., Zheng H., Lei S., Li Y., Zhang J. (2022). Dynamic Interaction Between Deforestation and Rice Cultivation During the Holocene in the Lower Yangtze River, China. *Front. Earth. Sci.* 10, 849501. doi: 10.3389/feart.2022.849501

He K., Lu H., Zheng H., Yang Q., Sun G., Zheng Y., Cao Y., Huan X. (2020). Role of dynamic environmental change in sustaining the protracted process of rice domestication in the lower Yangtze River. *Quat. Sci. Rev.* 242, 106456. doi: 10.1016/j.quascirev.2020.106456

He K., Lu H., Zheng Y., Zhang J., Xu D., Huan X., Wang J., Lei S. (2018). Middle-Holocene sea-level fluctuations interrupted the developing Hemudu culture in the lower Yangtze River, China. *Quat. Sci. Rev.* 188, 90-103. doi: 10.1016/j.quascirev.2018.03.034

Hu Y., Zhou B., Lu Y., Zhang J., Min S., Dai M., Xu S., Yang Q., Zheng H. (2020). Abundance and morphology of charcoal in sediments provide no evidence of massive slash-and-burn agriculture during the Neolithic Kuahuqiao culture, China. *PLoS One*. 15, e0237592. doi: 10.1371/journal.pone.0237592

Jiang L. (2014). *A study of Kuahuqiao culture*. Beijing: Science Press. (in Chinese)

Lei S., Ding F., Wu B. (2016). Excavation report of Phase I of the Yushan site in Zhenhai, Ningbo, Zhejiang. *Southeast Culture.* 18. (in Chinese)

Lei S., Wang J. (2016). Archaeological excavations at the Yushan and Wuguishan site in Zhenhai. *Popular Archaeology.* 32-41. (in Chinese)

Li C., Zheng Y., Yu S., Li Y., Shen H. (2012). Understanding the ecological background of rice agriculture on the Ningshao Plain during the Neolithic Age: pollen evidence from a buried paddy field at the Tianluoshan cultural site. *Quat. Sci. Rev.* 35, 131-138. doi: 10.1016/j.quascirev.2012.01.007

Liu Y., Deng L., He J., Jiang R., Fan D., Jiang X., Jiang F., Li M., Chen J., Chen Z., et al. (2020). Early to middle Holocene rice cultivation in response to coastal environmental transitions along the South Hangzhou Bay of eastern China. *Paleogeogr. Paleoclimatol. Paleoecol.* 555, 4, 109872. doi: 10.1016/j.palaeo.2020.109872

Liu Y., Sun Q., Fan D., Lai X., Xu L., Finlayson B., Chen Z. (2016). Pollen evidence to interpret the history of rice farming at the Hemudu site on the Ningshao coast, eastern China. *Quat. Int.* 426, 195-203. doi: 10.1016/j.quaint.2016.05.016

Ma C., Wang B., Liu Z., Zhao L., Hu Z., Sun G., Yang Q., Zhou Y., Zheng H. (2018). A preliminary study on vegetation, environment and human activity of pollen records during Middle-Late Holocene in Tianluoshan site, Ningshao Plain. *Quat. Sci.* 38, 1304-1312. doi: 10.11928/j.issn.1001-7410.2018.05.22 (in Chinese)

Ningbo Municipal Institute of Cultural Relics and Archaeology and Cixi Museum. (2012). Excavation Report of Tongjia 'ao Site, Cixi city, Zhejiang Province, 2009. *Southeast Culture*. 66-79. (in Chinese)

Ningbo Municipal Institute of Cultural Relics and Archaeology, Department of Archaeological Relics, School of History, Nanjing University, Ningbo Fenghua District Cultural Relics Protection and Management Institute (2019). Brief excavation of the Fangqiao excavation area of the Hejia site in Ningbo, Zhejiang. *Cultural Relics in Southern China* 01, 56-72. (in Chinese)

Ningbo Municipal Institute of Cultural Relics and Archaeology, Department of Archaeology and Museology, Renmin University of China, Ningbo Fenghua District Cultural Relics Protection and Management Institute. (2019). Brief excavation of the Fangqiao Excavation Area of the Xiawangdu Site in Ningbo, Zhejiang. *Archaeology*. 09, 3-16. (in Chinese)

Ningbo Municipal Institute of Cultural Relics and Archaeology. (2013). *Fujiashan: Excavation report of the Neolithic site*. Beijing: China Science Publishing. (in Chinese)

Official account of Qianjiang Evening News, *Archaeological Discoveries Top 10 in Zhejiang in the New Era——Shi'ao Site in Yuyao*, Qianjiang Evening News, 2022-06-02 (in Chinese)

Pan Y., Yuan J. (2018). Subsistence in the lower Yangtze River region from the neolithic to pre-qin period (part one). *Cultural Relics in Southern China*. 111-125. (in Chinese)

Shaoxing Ningbo Coastal Plain, East China. East China Normal University, Doctoral

Sun G., Huang W. (2000). *Excavation report of Xiangjiashan site in Yuyao City. In: Prehistory Culture*. Xi'an: San Qin Press. pp. 385-427. (in Chinese)

Tang, L. (2019). *Middle Holocene Sea Water Intrusion and Human’s Rapid Response in the Shaoxing Ningbo Coastal Plain, East China*. [dissertation/doctor’s thesis]. [Shanghai]: East China Normal University.

Wang J., Jiang L., Sun H. (2021). Early evidence for beer drinking in a 9000-year-old platform mound in southern China. *PloS one.* 16, e0255833. doi: http://dx.doi.org/10.1371/journal.pone.0255833

Wu Y., Li D., Sha L., Shu J., Wang W., Xu X. (2016). A diatom record of rapid sea-level rise during early to middle Holocene in Baima lake area, Zhejiang Province. *Acta. Palaeotologica. Sinica.* 55, 495-507. doi: 10.19800/j.cnki.aps.2016.04.010

Zhejiang Provincial Institute of Cultural Relics and Archaeology. (2016). *The Shangshan culture discoveries and narravites*. Beijing: Cultural Relics Publishing House. (in Chinese)

Zhejiang Provincial Institute of Cultural Relics and Archaeology, Xiangshan County Cultural Relics Management Committee. (2014). *Xiangshan and Tashan sites*. Beijing: Cultural relics publishing house. (in Chinese)

Zhejiang Provincial Institute of Cultural Relics and Archaeology, Department of History Xiamen University (2001). Excavation report of the Zishan site in Yuyao City, Zhejiang Province. *Archaeology*. 14-25. (in Chinese)

Zhejiang Provincial Institute of Cultural Relics and Archaeology, Ningbo Institute of Cultural Heritage Management, Yuyao Hemudu Site Museum (2021). Brief Report on the Excavatation of the Neolithic Site at Jingtoushan in Yuyao City, Zhejiang. *Archaeology*. 3-26. (in Chinese)

Zhejiang Provincial Institute of Cultural Relics and Archaeology. (2003). *Hemudu: A Neolithic site and its archaeological excavations*. Beijing: Cultural Relics Press. (in Chinese)

Zheng X., Lei S., Wang J., Zhijun Z. (2019). Analysis of Flotation Results from the Yushan Site in Ningbo City. *Agricultural Archaeology*. 21-27. (in Chinese)

Zheng Y., Sun G., Qin L., Li C., Wu X., Chen X. (2009). Rice fields and modes of rice cultivation between 5000 and 2500 BC in east China. *J. Archaeol. Sci.* 36, 2609-2616. doi: 10.1016/j.jas.2009.09.026

Zhu X., Dong N., Lei S. (2020). Research on fish remains excavated from the Wuguishan site in Zhenhai, Ningbo. *Cultural Relics in Southern China*. 15. (in Chinese)
